# Mechanical properties, degree of sclerotisation and elemental composition of the gastric mill in the red swamp crayfish *Procambarus clarkii* (Decapoda, Crustacea)

**DOI:** 10.1038/s41598-022-22724-w

**Published:** 2022-10-23

**Authors:** Wencke Krings, Jan-Ole Brütt, Stanislav N. Gorb

**Affiliations:** 1 Department of Behavioral Biology, Institute of Cell and Systems Biology of Animals, Universität Hamburg, Martin-Luther-King-Platz 3, 20146 Hamburg, Germany; 2 Department of Mammalogy and Paleoanthropology, Leibniz Institute for the Analysis of Biodiversity Change, Martin-Luther-King-Platz 3, 20146 Hamburg, Germany; 3 Department of Functional Morphology and Biomechanics, Zoological Institute, Christian-Albrechts-Universität Zu Kiel, Am Botanischen Garten 1-9, 24118 Kiel, Germany

**Keywords:** Structural biology, Biomechanics

## Abstract

The gastric mill of Decapoda is a unique feature, which comprises teeth, stabilizing ossicles, and particle sorting setae. Involved in the fragmentation and sorting of the food, this structure serves as interface between the organism and its environment. As material properties complement morphology and hold information about function and trophic preferences, we here provide a basis for more comparative research on gastric mills. For gastric mill components of the adult red swamp crayfish *Procambarus clarkii*, we studied (a) the micro-structure via scanning electron microscopy, (b) the elemental composition by energy-dispersive X-ray spectroscopy, (c) the heterogeneities in material properties and degree of tanning (autofluorescence) by confocal laser scanning microscopy, and (d) the mechanical properties hardness and elasticity by nanoindentation technique. The morphology and micro-structure were previously described for this species, but the mechanical properties and the autofluorescence were not studied before. As epicuticle and exocuticle could be analyzed individually, material property gradients, with values decreasing from the interacting surface towards interior, could be determined. Finally, we were able to relate the mechanical property data with the elemental composition and the degree of tanning. We found that the epicuticle of the teeth is among the hardest and stiffest biological materials in invertebrates due to the incorporations of high proportions of silicon.

## Introduction

### The gastric mill—a characteristic feature in decapod crustaceans

Within the Arthropoda, the crustaceans exhibit, with about 67,000 described species, an extraordinary diversity in body plans, which enabled them to colonialize almost all habitats^1^. This is accompanied by the ability to forage on a variety of food types with distinct mechanical properties^2,3^ and by the evolution of specialized appendages as mandibles, maxillae, or maxilipeds, which process the food mechanically^2,4–7^. The Decapoda, in addition, evolved a specialized and complex foregut, which comprises, besides the esophagus, the cardiac and the pyloric chamber of the stomach. The cardiac chamber stores the food intaken and additionally fragments the food mechanically. This is done by a complex assembly of stabilizing ossicles and interacting teeth, which were termed “gastric mill” [e.g.,^8–12^]. After fragmentation, food particles are transported into the pyloric chamber, passing the cardiopyloric valves, which function as barrier and masticatory ossicle^4^. The pyloric chamber is covered with setae, which allow only fine particles to reach the midgut glands and sort course ones to the hindgut.

With regard to the gastric mill components, excellent anatomical, morphological, and also elemental analyses were performed^13–16^. Additionally, the gastric mill was studied in the light of age determination^17–21^ and communication^22^. As this part of the stomach is morphologically diverse^23,24^, it triggered an ongoing discussion^25^ to which extent the shapes of the gastric mill components reflect adaptations to food items and feeding habits^4,10,26–32^ or phylogenetic constrains^4,5,33–35^.

Adaptations to food items are, however, not only reflected by the morphology of food-handling structures, but additionally by their material properties. Biological materials are the result of a long-lasting evolution and generally composites with heterogeneities or gradients, which contribute to the function [for review see^36^]. Knowledge about material properties complements morphology and gives rise to a profound understanding of function and also—when feeding structures are studied comparatively—of trophic specialisations. For invertebrate feeding structures, only very few studies comparing multiple taxa in the light of trophic specialisation implement both material properties and morphology [for fly teeth, see^37^; for butterfly proboscises, see^38^ and for radular teeth, see^39–43^.

### Aim of the study

In the following, we provide a basis for more comparative research on gastric mill material properties. We present data on the epi- and exocuticle of gastric mill components from adult red swamp crayfish *Procambarus clarkii* (Girard, 1852) (Decapoda, Crustacea). This species forages rather herbivorous in pre-adult and adult stage and carnivorous in juvenile stage^44^. Previously, the morphology, micro-structure, and elemental composition of the gastric mill from this species was studied during intermolt stage^13^, but data on the (a) mechanical properties (hardness and Young’s modulus) and (b) the local composition of the chitin layers is lacking.

(A) First, we studied the micro-structure of the gastric mill components to identify epi-, exo-, and endocuticle. This was done by fragmentation of the components and subsequent careful examination under the scanning electron microscope (SEM). (B) Then, we applied energy-dispersive X-ray spectroscopy (EDS, EDX) to determine the elemental composition of the epi- and exocuticle in the targeted structures. These analyses were performed to determine the origins of the heterogeneities in the mechanical properties hardness and elasticity (i.e., Young’s modulus), that were identified by nanoindentation technique (C). (D) We additionally visualized the structures by confocal laser scanning microscopy (CLSM) with four lasers of different wavelength. The protocol used was previously applied to various arthropod structures and could relate the autofluorescence signal to the degree of tanning and the organic content. Finally, we were able to relate the mechanical property data and the autofluorescence signals of the cuticle with the elemental composition and the degree of tanning.

## Materials and methods

### Samples

The animals were collected from freshwater bodies in Berlin, Germany, in 2021 in the course of invasive alien species management, based on the updated EU Regulation 1143/2014. They were killed, fixed in 70% EtOH and acquired by the Department of Biology (Universität Hamburg) for the annual dissection class. Here, adult specimens of similar size (11–12 cm) were dissected by the students, the intact stomachs extracted and stored again in 70% EtOH. 18 stomachs were selected for this study. The surrounding tissues were carefully removed, the stomachs opened, cleaned by a short ultrasonic bath, and finally stored in EtOH.

### Documentation and terminology

**Figure 1:**
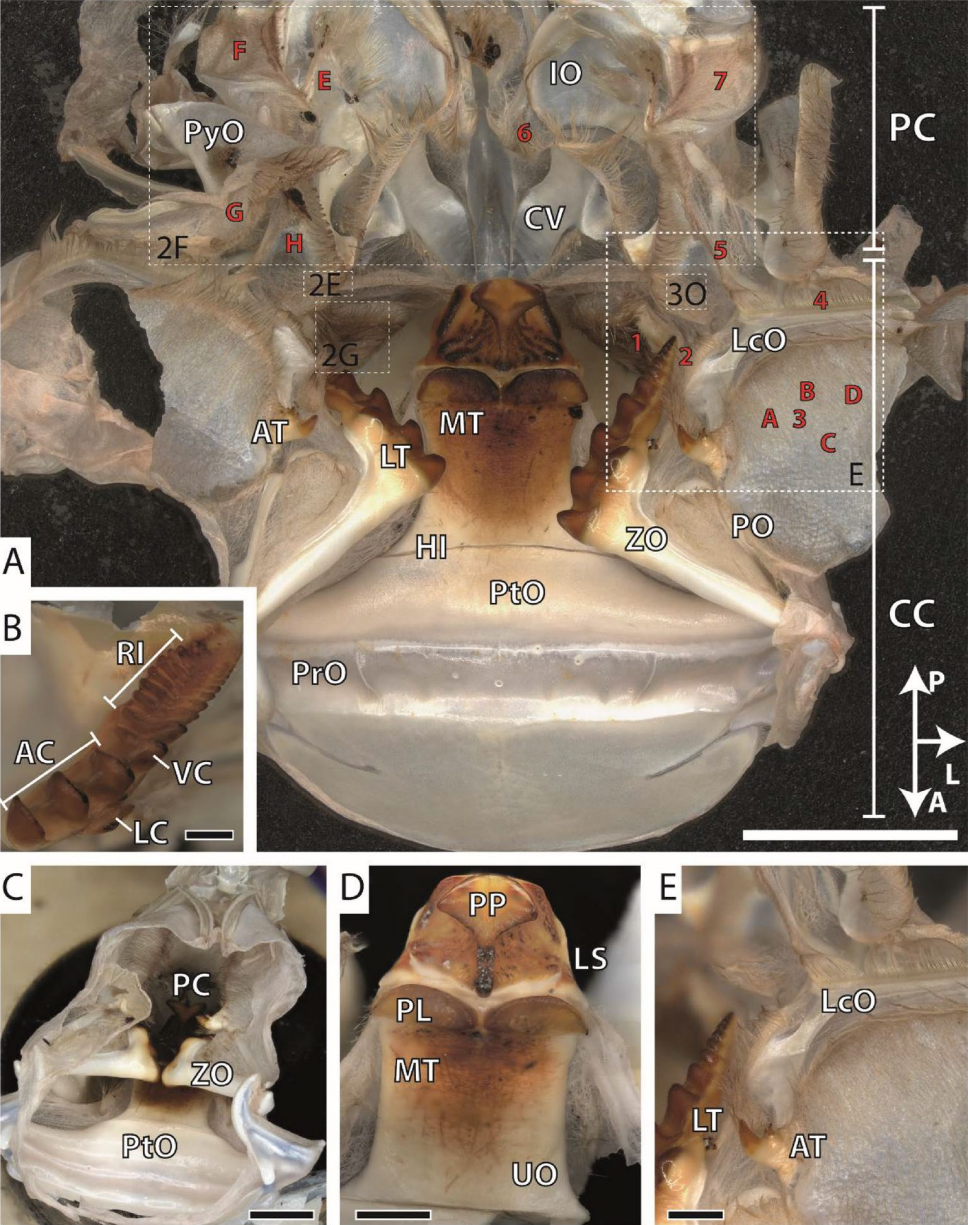
Light microscopy images from the gastric mill of *Procambarus clarkii*. (**A**) Dissected gastric mill (red letters highlight the localities of the tested membrane and red numbers—the localities of the tested setae; the arrow points in the anterior, posterior, and lateral directions. (**B**) Lateral tooth. (**C**) Gastric mill with interacting lateral teeth. (**D**) Medial tooth. (**E**) Accessory tooth. *A* anterior, *AC* anterior cusp of the lateral tooth, *AT* accessory tooth, *CC* cardiac chamber, *CV* cardiopyloric valve, *HI* hinge of medial tooth, *IO* inferior ampullary ossicle, *L* lateral, *LC* lateral cusp of the lateral tooth, *LcO* lateral cardiac ossicle, *LS* lateral spines of medial tooth, *LT* lateral tooth, *M* membrane, *MT* medial tooth, *P* posterior, *PC* pyloric chamber, *PL* plate of the medial tooth, *PO* pectineal ossicle, *PP* posterior process of the medial tooth, *PrO* prepterocardiac ossicle, *PtO* pterocardiac ossicle, *PyO* pyloric ossicle, *RI* ridges of the lateral tooth, *S* setae, *UO* urocardiac ossicle, *VC* ventral cusp of the lateral tooth, *ZO* zygocardiac ossicle. Scale bars: A, 3000 µm; B, 400 µm; C, 2000 µm; D, 1 mm; E, 800 µm.

We focus in this study on overall nine gastric mill structures: the accessory tooth, the cardiopyloric valve, the lateral tooth, the lateral cardiac ossicle, the medial tooth, the membrane from different stomach areas, the pterocardiac ossicle, the setae from different areas, and the zygocardiac ossicle. Overall, we studied 18 stomachs; all of them were first documented by light microscopy, employing a Keyence Digital Microscope VHX-7000 (KEYENCE, Neu-Isenburg, Germany) equipped with automatic stacking software (Fig. 1). Structures were termed according to the previous literature^14,45–48^ (see Fig. 1). Parts (e.g., medial tooth, lateral tooth, ossicles, etc.) of five additional stomachs were fragmented by stiff tweezers to study the layered organization of the cuticle by light microscopy (Supplementary Fig. 1).

**Figure 2:**
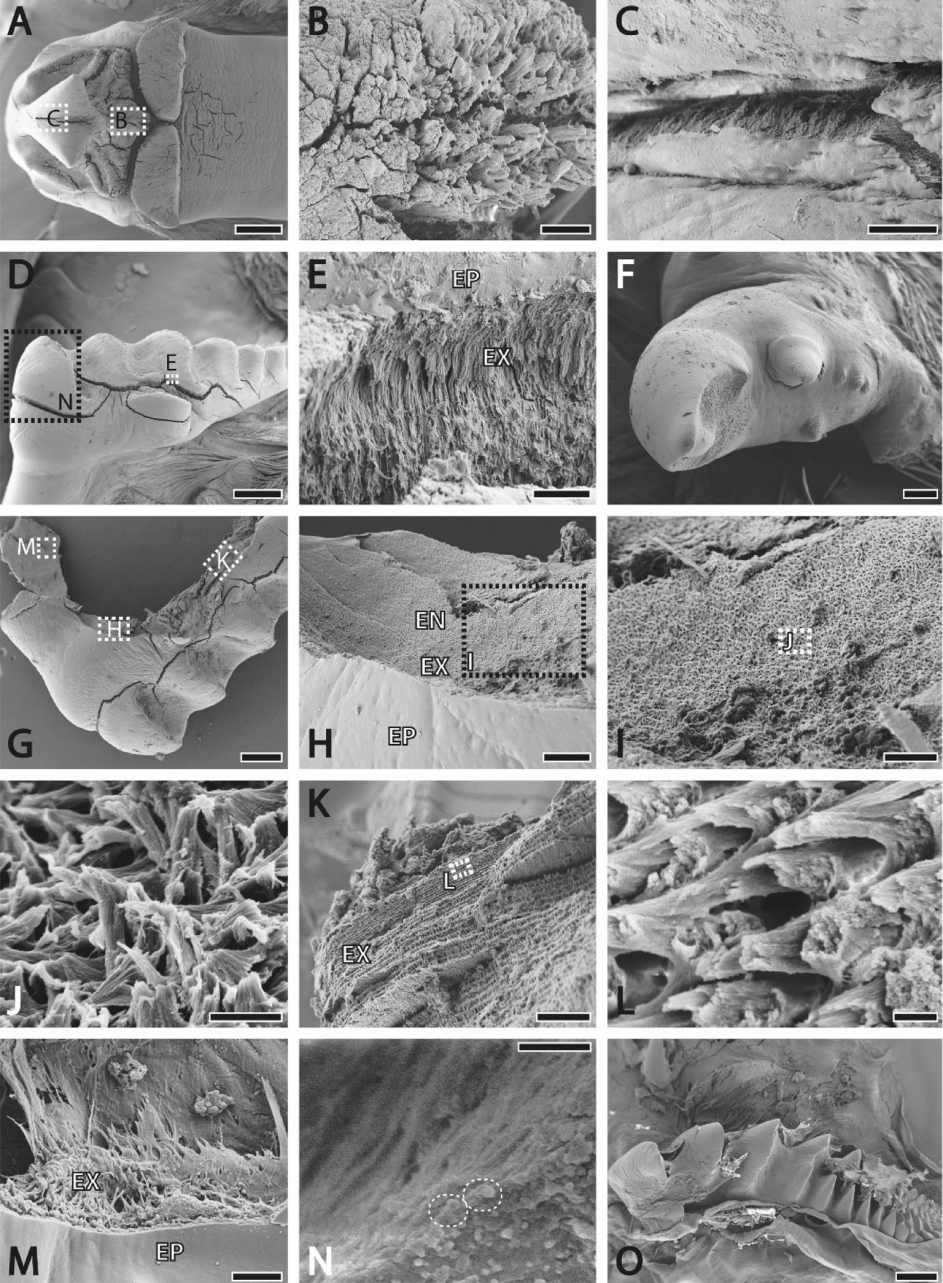
SEM images. (**A**)–(**C**) Medial tooth with magnifications of the tooth surface (**B**–**C**). (**D**)–(**E**) Lateral tooth and its fracture at high magnification (**E**) of the epicuticle and the fibrous exocuticle. (**F**) Accessory tooth. (**G**)–(**M**) Chitin fiber structure of the lateral tooth basis. (**J**) Fibers are bend helically. (**K**)–(**L**) Layered structure of the exocuticle at low and high magnifications (**L**). M. Chitin fiber orientation in the exocuticle. (**N**) Fractured medial tooth with potential fracture artefacts between fibers. (**O**) Immature lateral tooth. *EN* endocuticle, *EP* epicuticle, *EX* exocuticle. Scale bars: A, 800 µm; B–C, H, 30 µm; D, 600 µm; E, I, 20 µm; F, 100 µm; G, 400 µm; J, 2 µm; K, 30 µm; L, 600 nm; M, 10 µm; N, 1 µm; O, 400 µm.

**Figure 3:**
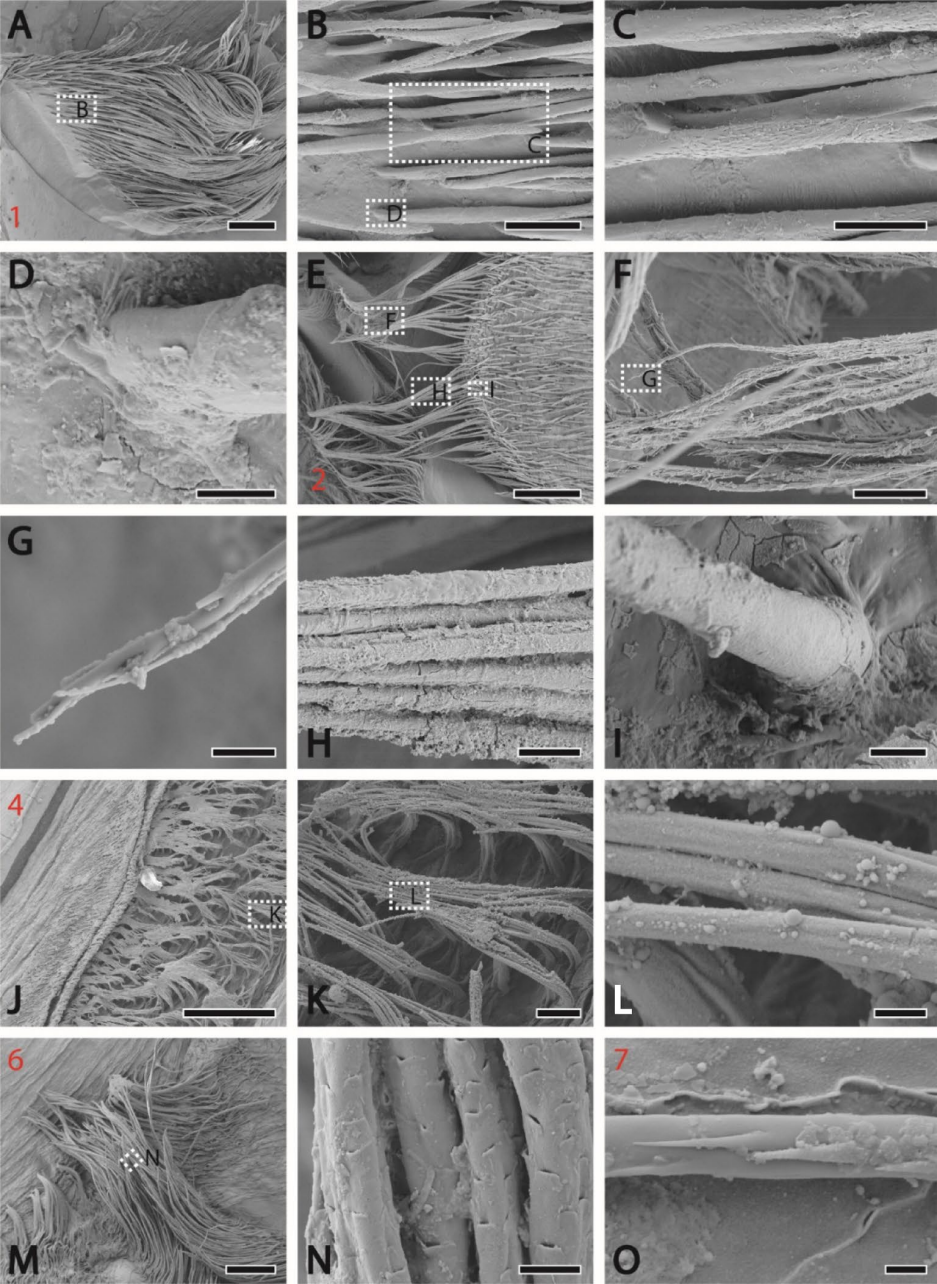
SEM images of the setae from distinct localities (the locality number is highlighted in red). (**A**) Presumably cuspidate setae with bold shaft and tip bearing small projections from the locality 1 with magnifications (**B**)–(**D**). (**D**, **I**) Attachment of the setae with the membrane; no socket could be seen. (**E**) Setae [also at high magnifications (**F**)–(**I**)] bearing long serrated setules (plumodenticulate setae), which agglomerated and formed a net, from the locality 2. (**J**) From the locality 4, long setae [with magnifications (**K**)–(**L**)] without setules of denticles, clustering together forming mattes. (**M**) From the locality 6; long setae [at high magnification (**N**)] bearing small scales (pappose setae). (**O**) Setae with small projections on the tip from the locality 7. Scale bars: A, E, 400 µm; B, 80 µm; C, F, 40 µm; D, 8 µm; E, H, K, 20 µm; F, 100 µm; G, L, 3 µm; I, N, 6 µm; J, 200 µm; K, 20 µm; M, 200 nm; O, 2 µm.

After light microscopy, the components of the fragmented stomachs and of six intact ones were studied by SEM (Figs. 2, 3). For SEM images, structures were arranged on SEM sample holders, sputter-coated with platinum (5 nm layer) and documented with the Zeiss LEO 1525 (One Zeiss Drive, Thornwood, USA) to receive images with a high resolution.

### Confocal laser scanning microscopy (CLSM)

For arthropod cuticle, the laser excitation via CLSM according to the protocol of^49^, allowed the identification of regions with the following material compositions: sclerotized, stiff cuticle is associated with a red signal and weakly-sclerotized chitin with a green one. Blue signals were previously identified as regions with high proportions of resilin or related proteins.

**Figure 4:**
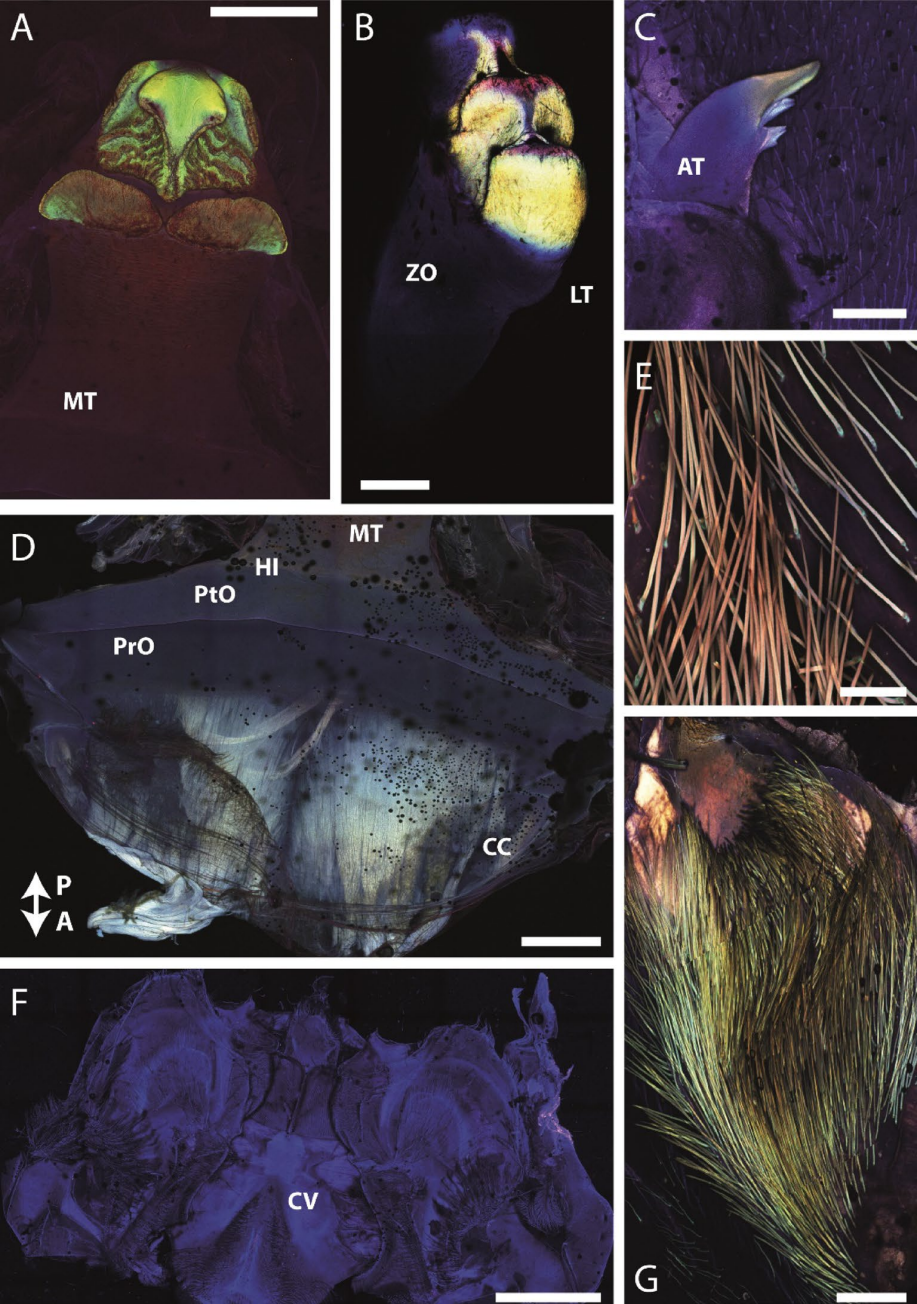
CLSM images of the components of the gastric mill. Comparison of colors is only possible within each individual image, as they were taken individually. (**A**) Medial tooth. (**B**) Lateral tooth. (**C**) Accessory tooth. (**D**) Basis of medial tooth and cardiac chamber. The arrow points in the anterior and posterior direction. (**E**). Setae with high magnification. (**F**) Cardiopyloric valve and surrounding structures. (**G**) Setae and surrounding membrane. *A* anterior, *AT* accessory tooth, *CC* cardiac chamber, *CV* cardiopyloric valve, *HI* hinge of medial tooth, *LT* lateral tooth, *MT* medial tooth, *P* posterior, *PrO* prepterocardiac ossicle, *PtO* pterocardiac ossicle, *ZO* zygocardiac ossicle. Scale bars: A, 750 µm; B, 200 µm; C, 400 µm; D, 750 µm; E, 20 µm; F, 1500 µm; G, 100 µm.

To identify the autofluorescence (Fig. 4), five additional stomachs and their components were used. They were first rinsed with water, arranged on glass slides and surrounded with modelling clay to avoid contact between sample and cover slip. Glycerin (greater than or equal to 99.5%, free of water, Carl Roth GmbH & Co. KG, Karlsruhe, Germany) was placed onto the stomachs until it was completely covered. Finally, a glass slip was deposited on each sample.

Stomachs were visualized employing a Zeiss LSM 700 confocal laser scanning microscope (Carl Zeiss Microscopy GmbH, Jena, Germany) following the protocol of^49^. To visualize the autofluorescence, four stable solid-state lasers were used (wavelengths of 405 nm, 488 nm, 555 nm, and 639 nm). To capture the emitted autofluorescence, we applied bandpass or long pass emission filters, transmitting light of wavelengths 420–480 nm, greater than or equal to 490 nm, greater than or equal to 560 nm, and greater than or equal to 640 nm. Objective lenses with × 5 (Zeiss Plan-Apochromat, numerical aperture (NA) = 0.16), × 10 (Zeiss EC Plan-Neofluar, NA = 0.45), or × 20 (Zeiss Plan-Apochromat, air immersion, NA = 0.8) magnification were applied. Colors, blue, green, red (50% saturation) and red (50% saturation) were assigned to each image. Subsequently, maximum intensity projection was calculated, employing the software Zeiss Efficient Navigation (Zen) (Carl Zeiss Micro Imaging GmbH).

### Energy-dispersive x-ray spectroscopy (EDX) and nanoindentation

By EDX, the local elemental composition can be determined. Nanoindentation technique allows the identification of the local mechanical properties hardness (H) and Young’s modulus (E, elasticity, elastic modulus). EDX and nanoindentation analyses were performed on the same two additional gastric mills. Since we worked with embedded samples (see below), we could perform these two tests always at the same localities—EDX analyses were performed first and afterwards the same site was tested by nanoindentation.

We tested the epicuticle, which can be identified by the lack of fibers, and the exocuticle in most structures. We did not study the endocuticle, because the outer layer (epi- plus exocuticle) has an intimate interaction with the food and could thus show adaptations to trophic preferences. The significantly softer endocuticle, in contrast, probably contributes to function by suppressing micro cracks, as shown by previous studies on crustaceans^50–56^.

By EDX and nanoindentation, we could test the epi- and exocuticle of the accessory tooth, the cardiopyloric valve, the lateral tooth, the lateral cardiac ossicle, the medial tooth, the pterocardiac ossicle, and the zygocardiac ossicle at different localities (for the cuticle layers, see Supplementary Fig. 1). Due to the thinness of the membrane and the setae, only the elemental composition was studied without clear discrimination between epi- and exocuticle. In the membrane, the discrimination between epi- and exocuticle was rather difficult; as a result, the “membrane epicuticle” of this study could be “epicuticle and some layers of the exocuticle”. For each seta, we could differentiate between basis and tip, but did not sort the results to the cuticle layer.

Stomachs were attached to glass object slides (Carl Roth, Karlsruhe, Germany) with double-sided adhesive tape. After drying at room temperature, each stomach was surrounded by a metallic ring, which was filled with epoxy resin (RECKLI EPOXI WST, RECKLI GmbH, Herne, Germany), polymerizing for three days at room temperature (Young’s modulus of the polymerized epoxy is 1.3 ± 0.3 GPa). The metallic ring ensured, that the sample surface was almost parallel to the sample holder. This specific epoxy was chosen, because, known from previous studies^39–43,57,58^, it does not infiltrate the structures. After polymerization, object slide and adhesive tape were removed and each sample was polished with sandpapers of distinct roughness until the structures targeted were on display. Then, the surface was smoothened on a polishing machine (Minitech 233/333, PRESI GmbH, Hagen, Germany) with aluminum oxide polishing powder suspension of 0.3 μm grainsize (PRESI GmbH, Hagen, Germany). Samples were cleaned in an ultrasonic bath for five minutes, dried, and sputter-coated with platinum (5 nm layer). By this methodology we received sections of the structures, which allowed the testing of the epicuticle and exocuticle—in most cases.

Before analyzing a sample by EDX, the detector was always calibrated with copper (Cu). As it could not be calibrated with H (hydrogen) additionally, our results are semi-quantitative, i.e. the results on the proportions of lighter elements are not as reliable as the same of heavier elements. For the semi-quantitative EDX, we employed the SEM Zeiss LEO 1525 (One Zeiss Drive, Thornwood, New York, USA) equipped with an Octane Silicon Drift Detector (SDD) (micro analyses system TEAM, EDAX Inc., New Jersey, USA) [for a detailed protocol see^58–62^]. For all measurements, we used an acceleration voltage of 20 keV and the same settings (e.g., lens opening, working distance, etc.). We tested smaller areas with sizes ranging from 4 × 3 µm (for setae) up to 20 × 15 µm (for epicuticle), to receive reliable results (Supplementary Fig. 1).

Aluminum (Al), carbon (C), calcium (Ca), chloride (Cl), fluorine (F), hydrogen (H), iron (Fe), potassium (K), magnesium (Mg), sodium (Na), oxygen (O), phosphorus (P), platinum (Pt), sulfur (S), silicon (Si), and zinc (Zn) were detected, and their proportions measured. We used the data of atomic ratio (atomic %) for this study. Values were received with two positions after the decimal point, but were rounded to one decimal point, because of detection limits. We do not discuss the following elements, because they are either the elemental basis of chitin (C, H, O), the coating (Pt), or of the polishing powder (Al, O). For some calculations, Ca, Cl, F, Fe, K, Mg, Na, P, S, Si, and Zn were summarized as “all elements” (Ae).

For nanoindentation [for a detailed protocol see^39,42,43,57,58,61^], a nanoindenter SA2 (MTS Nano Instrument, Oak Ridge, Tennessee, USA) equipped with a Berkovich indenter tip and a dynamic contact module (DCM) head was employed. A Poisson’s ratio of 0.3 was used. Hardness (H) and Young’s modulus (E) were determined from force-distance curves by applying the continuous stiffness measurement technique^63^. All tests were performed under normal room conditions (relative humidity 28–30%, temperature 22–24 °C) and each indent and its curve were manually controlled. Mechanical properties were determined at penetration depth of 480–520 nm. For each locality, we received 30 values, which were averaged to receive one mean value per indent. After testing the targeted region, the samples were polished until the next region of interest was on display.

All steps of the protocol (EDX and nanoindentation) were repeated until all localities were tested (for localities, see Supplementary Figs. 7, 8, 9, 10, 11, 12, 13). Overall, 221 localities in two stomachs were studied.

### Statistical analyses

Statistical analyses were performed with JMP Pro, Version 14 (SAS Institute Inc., Cary, North Caroline, 1989–2007). Mean values and standard deviations were calculated and Shapiro–Wilk-W-tests for testing of normality were conducted. When the data was normally distributed, t-tests were carried out; when it was not-normally distributed Chi-Square tests, followed by pairwise comparison with Wilcoxon method, were conducted. Correlation coefficients were also computed with JMP software.

## Results

### Morphology

For light microscopic images see Fig. 1; for SEM images see Figs. 2, 3.

The medial tooth possesses a prominent posterior process, which bears two rounded projections pointing towards lateral (for directions see Fig. 1). Below this processus, several smaller projections run from medial to lateral. Anteriorly, two roundish and prominent plates with sharp edges are found. The medial tooth interacts with the broad and thick anterior pterocardiac ossicle through a thin hinge. The pterocardiac ossicle is fused anteriorly with the thin prepterocardiac ossicle, which is merged with the thin cardiac chamber. Each lateral tooth bears three roundish and large anterior cusps and one smaller ventral cusp. Ten ridges, decreasing in thickness towards posterior, are found. On the outer side of each lateral tooth, one additional cusp is located. The bases of these teeth merge with the zygocardiac ossicle, which interacts with the pterocardiac ossicle through a joint, pointing towards lateral. The accessory tooth contains one prominent denticle and three smaller projections. Anteriorly, the accessory tooth interacts with the thin, brace-like pectineal ossicle and posteriorly with the thicker lateral cardiac ossicle. Pectineal ossicle, together with zygocardiac ossicle, pterocardiac ossicle, and cardiac ossicle seems to function as stabilators, preventing the stomach from collapsing during feeding.

Between the ossicles, the cuticle is membranous, softer, and densely packed with setae (Fig. 3). Depending on the locality, setae shows distinct morphologies of the shaft, whereas their attachment to the membrane seems to be of similar morphology; we could not identify sockets, but a rather direct transition from membrane to setae bases. In locality 1, half of the seta shaft (the basis) is free of setules or denticles; only the tips bear small projections (Fig. 3A–D). Locality 3 is covered with setae with longer projections. Locality 2 is covered with long setae bearing long serrated setules (plumodenticulate setae), which are agglomerated and seem to form a net (Fig. 3E–I). Localities 4 and 5 bear long setae without setules or denticles, clustering and forming mattes (Fig. 3J–L).

The voluminous cardiac chamber merges posteriorly with the small pyloric chamber; both chambers are connected by the cardiopyloric valves. The pyloric chamber is stabilized by the pyloric ossicles and inferior ampullary ossicles; its inner membrane is covered with setae (localities 6 and 7). Locality 6 bears long setae covered with small scales (potentially pappose setae; Fig. 3M–N) and locality 7 setae similar to those from locality 3 (Fig. 3O). As the stomach is molted together with the carapace, immature structures could be found underneath the cuticle in some specimens (Fig. 2O).

### Micro-structure

Even without damaging the teeth, deep cracks in epi- and exocuticle, running along the fibers, appeared as an artefact from drying at room temperature (Fig. 2). This already indicates, that teeth are not homogeneous but rather composed of layers with distinct material properties.

The epicuticle could be identified by the lack of chitin fibers (Fig. 2), in contrast to the exocuticle consisting of fibers. Between these fibers, larger aggregations of particles (size ~ 0.3–0.6 µm) could be determined in the teeth and ossicles (Fig. 2N). They are probably artefacts from fracturing, but could potentially be also interpreted as crystals. With regard to fiber orientation, structures are heterogeneous: in the membrane of the cardiac sac, fibers run parallel to the surface. In the ossicles and teeth, however, they run perpendicular to the outer edges, then change their orientations more centrally (Figs. 2C,E,M). Here, the fiber bundles run parallel to the membrane and form neatly stacked layers. Each layer slightly changes orientation (Fig. 2K), resulting in a general helicoidal structure (Figs. 2I,J).

### Autofluorescence

When the stomach was examined in one CLSM scan, the medial teeth exhibited such a strong autofluorescence that—after adapting the settings—we could not detect signals from other structures.

When each structure was studied individually, heterogeneities within the structures could be visualized. The medial tooth (the prominent posterior process, its projections and the two roundish plates) emitted a strong green autofluorescence (Fig. 4A). The lateral edges of the posterior process and the regions antero-medial to the plates appeared rather red. The remaining areas emitted a blue autofluorescence. In the lateral tooth, the anterior three cusps exhibited such a strong autofluorescence signal, that the remaining regions could not be visualized (Fig. 4B). A thin region, surrounding the bases of the anterior cusps, appeared blue, whereas most of the lateral tooth cusps seemed green. The tips of the three anterior cusps were red. In the accessory tooth (Fig. 4C), the prominent denticle emitted a green and the basis and the projections a blue autofluorescence signal. The cardiac and the pyloric chambers in general were blue without clear gradients (Figs. 4D,F). The hinge between the cardiac chamber and the pterocardiac ossicle exhibited a more intense blue signal. With high magnification, the membrane, embedding the setae, appeared blue; the setae tips emitted a red and the bases a green signal (Fig. 4E). When more of the surrounding membrane was included into the individual scan, the setae, however, appeared green (Fig. 4G). This depicts, that the membrane, in general, was rather heterogeneous in material composition.

### Mechanical properties

For the gastric mill, the values of H corresponded to the values of E (correlation coefficient of 0.99; see Supplementary Table 3).

**Figure 5:**
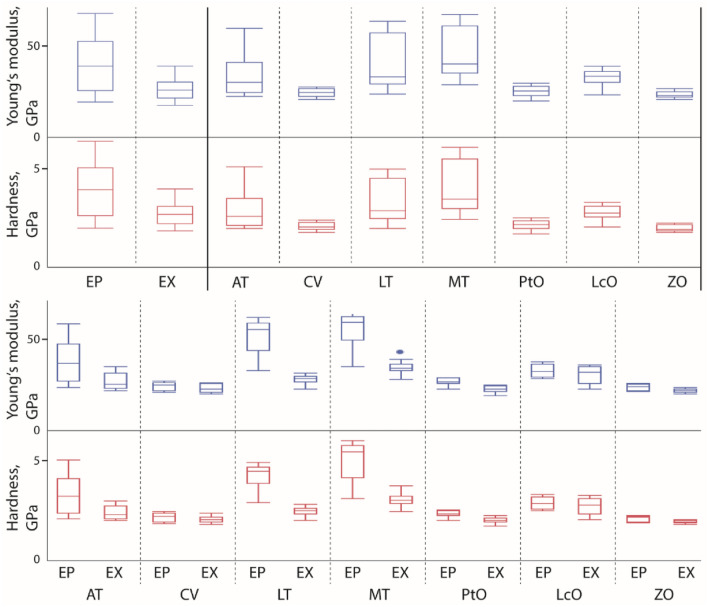
Hardness and Young’s modulus (both in GPa) of the distinct structures and regions tested are shown (for exact values, see Tables 1, 2 and Supplementary Table 1). Above *– left:* mechanical properties of epicuticle and exocuticle (data for all tested structures are pooled together). Above *– right:* mechanical properties of epicuticle and exocuticle are pooled together for AT, CV, LcO, LT, MT, PtO, and ZO. Below: mechanical properties of epicuticle and exocuticle separately presented for each structure tested (AT, CV, LT, MT, PtO, LcO, and ZO). *AT* accessory tooth, *CV* cardiopyloric valve, *EP* epicuticle, *EX* exocuticle, *LcO* lateral cardiac ossicle, *LT* lateral tooth, *MT* medial tooth, *M* membrane, *PtO* pterocardiac ossicle, *S* setae, *ZO* zygocardiac ossicle.

**Figure 6:**
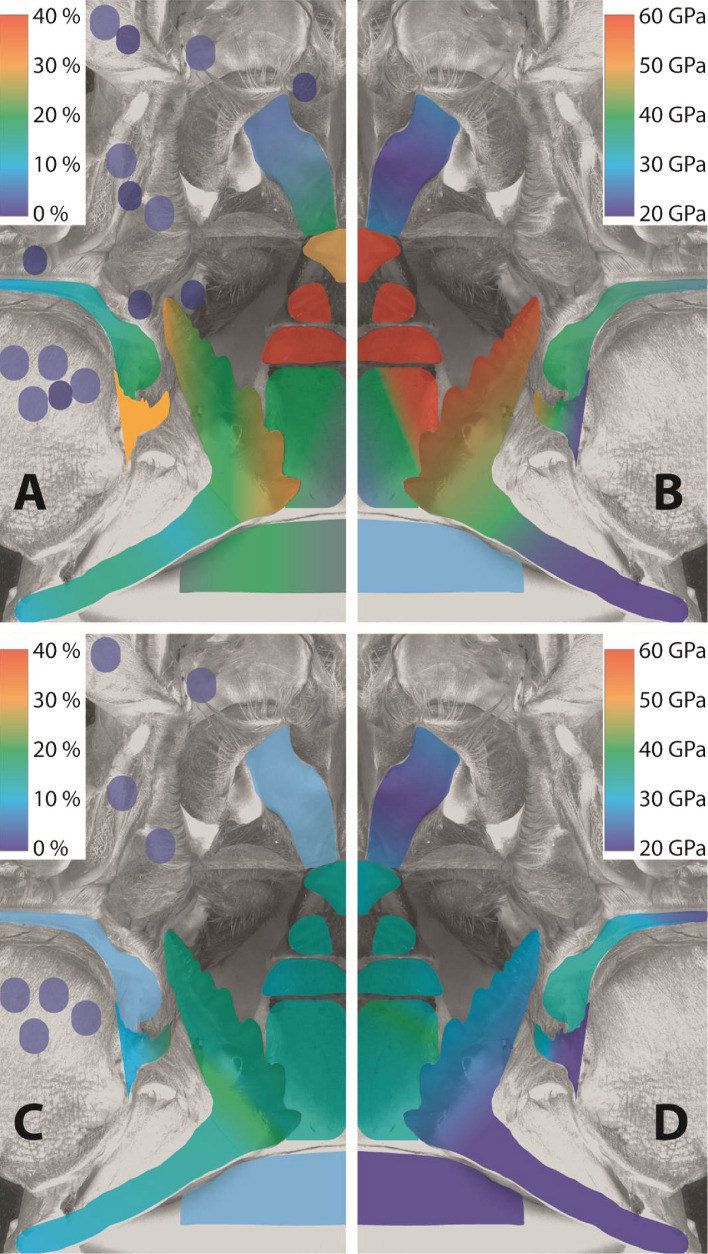
*Left side:* distribution of all elements (Ae), which are not the basis of chitin, in the epicuticle (above) and the exocuticle (below). *Right side:* Young’s modulus of the epicuticle (above) and the exocuticle (below). For the material property gradients, we used the mean values of the tested localities, rounded them to either 0%, 10%, 20%, 30%, and 40% for the elemental composition and to 20 GPa, 30 GPa, 40 GPa, 50 GPa, and 60 GPa for the Young’s modulus. The smallest value (0% and 20 GPa) was assigned to the color dark blue and the highest value (40% and 60 GPa) to red. These colors were then assigned to the localities tested by creating areas in illustrator, which were colored employing the illustrator gradient tool.

Our values of the E ranged from 20 GPa, measured in e.g. the exocuticle of the zygocardiac ossicle, up to 60 GPa, determined for the epicuticle of the lateral tooth cusps, the medial tooth cutting plates and projections (see Figs. 5, 6 and Table 1).

**Table 1:** The mean and SD of the mechanical properties (H and E both in GPa) and elemental proportions (in atomic %) for the epi- and exocuticle of each individual structure.

Structure	Cuticle layer		Ae	Ca	Cl	F	Fe	H	K	Mg	Na	P	S	Si	E	Zn
AT, accessory tooth	EP	Mean	21.12	6.81	1.01	1.48	0.40	3.32	0.12	0.43	0.08	3.82	0.18	5.68	38.22	0.22
		SD	6.49	1.17	0.32	0.58	0.14	1.04	0.07	0.15	0.14	0.81	0.07	5.83	12.00	0.06
		N	8	8	8	8	8	8	8	8	8	8	8	8	8	8
	EX	Mean	13.93	6.89	0.83	0.56	0.39	2.39	0.14	0.45	0.00	3.32	0.19	0.03	26.99	0.22
		SD	1.31	0.95	0.19	0.25	0.09	0.36	0.05	0.14	0.01	0.61	0.06	0.05	4.83	0.06
		N	8	8	8	8	8	8	8	8	8	8	8	8	8	8
CV, cardiopyloric valve	EP	Mean	15.33	5.38	0.61	3.24	0.52	2.16	0.12	0.65	0.00	2.98	0.35	0.01	24.51	0.53
		SD	2.46	1.40	0.15	0.28	0.13	0.23	0.03	0.15	0.00	0.93	0.13	0.01	2.27	0.35
		N	6	6	6	6	6	6	6	6	6	6	6	6	6	6
	EX	Mean	11.41	3.96	0.45	2.13	0.31	2.05	0.11	0.62	0.00	2.35	0.37	0.00	23.29	0.29
		SD	0.87	0.71	0.06	0.25	0.05	0.18	0.06	0.10	0.01	0.42	0.10	0.00	2.36	0.10
		N	6	6	6	6	6	6	6	6	6	6	6	6	6	6
LT, lateral tooth	EP	Mean	23.15	6.92	1.47	2.03	0.37	4.26	0.14	0.41	0.00	3.85	0.14	6.74	51.78	0.22
		SD	5.19	1.67	0.55	0.43	0.10	0.61	0.08	0.16	0.00	0.91	0.06	4.63	9.16	0.09
		N	22	22	22	22	22	22	22	22	22	22	22	22	22	22
	EX	Mean	15.30	7.25	1.42	1.77	0.35	2.47	0.10	0.34	0.00	2.87	0.13	0.00	28.15	0.20
		SD	2.21	1.01	0.39	0.41	0.11	0.22	0.07	0.11	0.00	1.87	0.06	0.01	2.17	0.07
		N	22	22	22	22	22	22	22	22	22	22	22	22	22	22
MT, medial tooth	EP	Mean	25.84	7.52	1.33	2.74	0.66	5.02	0.13	0.39	0.00	2.44	0.16	9.02	56.16	0.46
		SD	11.61	1.84	0.43	0.80	0.27	0.92	0.06	0.23	0.00	1.33	0.09	8.50	8.69	0.37
		N	22	22	22	22	22	22	22	22	22	22	22	22	22	22
	EX	Mean	15.18	6.19	1.36	2.26	0.60	3.05	0.10	0.43	0.00	2.50	0.17	0.00	34.81	0.52
		SD	1.25	0.86	0.45	0.76	0.18	0.27	0.07	0.22	0.00	1.02	0.09	0.01	3.05	0.27
		N	22	22	22	22	22	22	22	22	22	22	22	22	22	22
M, membrane	EP	Mean	1.54	0.06	0.10	0.07	0.00	–	0.03	0.09	0.00	0.10	0.21	0.00	–	0.01
		SD	0.35	0.10	0.08	0.15	0.00	–	0.03	0.07	0.00	0.11	0.17	0.00	–	0.02
		N	12	12	12	12	12	–	12	12	12	12	12	12	–	12
	EX	Mean	1.60	0.08	0.11	0.09	0.00	–	0.11	0.11	0.00	0.08	0.10	0.00	–	0.00
		SD	0.26	0.13	0.12	0.07	0.00	–	0.07	0.09	0.00	0.08	0.10	0.00	–	0.01
		N	12	12	12	12	12	–	12	12	12	12	12	12	–	12
PtO, pterocardiac ossicle	EP	Mean	14.90	6.82	1.01	1.12	0.37	2.34	0.12	0.50	0.03	3.51	0.23	0.04	26.93	0.26
		SD	1.98	1.18	0.39	0.29	0.16	0.18	0.08	0.35	0.04	0.82	0.12	0.04	2.03	0.09
		N	8	8	8	8	8	8	8	8	8	8	8	8	8	8
	EX	Mean	9.64	3.64	0.80	0.67	0.21	2.01	0.11	0.59	0.00	2.10	0.29	0.01	22.71	0.26
		SD	0.48	0.38	0.14	0.28	0.09	0.15	0.04	0.21	0.01	0.28	0.09	0.01	1.95	0.05
		N	8	8	8	8	8	8	8	8	8	8	8	8	8	8
LcO, lateral cardiac ossicle	EP	Mean	11.68	3.80	0.93	2.42	0.17	2.87	0.15	0.63	0.00	2.25	0.27	0.00	33.00	0.28
		SD	3.03	1.39	0.49	0.73	0.20	0.31	0.06	0.18	0.00	0.76	0.12	0.00	3.61	0.10
		N	8	8	8	8	8	8	8	8	8	8	8	8	8	8
	EX	Mean	10.70	3.30	0.81	2.21	0.22	2.74	0.06	0.54	0.00	2.02	0.29	0.00	30.97	0.34
		SD	1.37	0.71	0.22	0.72	0.17	0.42	0.07	0.15	0.00	0.65	0.08	0.00	5.02	0.10
		N	8	8	8	8	8	8	8	8	8	8	8	8	8	8
S, setae	Basis	Mean	1.13	0.14	0.01	0.04	0.00	–	0.01	0.01	0.00	0.04	0.00	0.00	–	0.01
		SD	0.13	0.10	0.02	0.04	0.00	–	0.02	0.01	0.00	0.05	0.01	0.00	–	0.01
		N	14	14	14	14	14	–	14	14	14	14	14	14	–	14
	Tip	Mean	1.78	0.51	0.00	0.15	0.00	-	0.00	0.03	0.00	0.1	0.03	0.00	–	0.00
		SD	0.35	0.15	0.00	0.10	0.00	-	0.01	0.05	0.00	0.09	0.03	0.00	–	0.00
		N	14	14	14	14	14	–	14	14	14	14	14	14	–	14
ZO, zygocardiac ossicle	EP	Mean	12.71	5.25	1.23	1.08	0.47	2.10	0.11	0.35	0.02	2.83	0.14	0.05	23.88	0.28
		SD	2.39	1.59	0.59	0.27	0.16	0.15	0.08	0.15	0.03	0.85	0.06	0.05	1.67	0.17
		N	6	6	6	6	6	6	6	6	6	6	6	6	6	6
	EX	Mean	10.25	4.29	0.69	0.76	0.45	1.93	0.10	0.41	0.00	2.23	0.17	0.00	21.87	0.25
		SD	0.89	0.77	0.26	0.18	0.06	0.07	0.06	0.17	0.00	0.54	0.07	0.01	1.13	0.08
		N	6	6	6	6	6	6	6	6	6	6	6	6	6	6

*E* Young’s modulus, *EP* epicuticle, *EX* exocuticle, *H* hardness, *N* quantity of tests, *SD* standard deviation.

Differences between epicuticle and exocuticle were highly significant for both H and E (see Table 2). The epicuticle was, in most structures, harder and stiffer than the exocuticle (see Figs. 5, 6 and Supplementary Table 1). Only in the cardiopyloric valve, the epi- and exocuticle were rather similar in their mechanical properties (see Figs. 5, 6 and Supplementary Table 1). The individual structures differed significantly in H and E values (Supplementary Table 2). The hardest and stiffest structure was the medial tooth, followed by lateral tooth, accessory tooth, lateral cardiac ossicle, pterocardiac ossicle, cardiopyloric valve, and finally the zygocardiac ossicle with the lowest values in H and E (see Fig. 5 and Supplementary Table 1).

**Table 2:** *Left:* The mean and SD of the mechanical properties (H and E, both in GPa) and elemental proportions (in atomic %) for the epi- and exocuticle (all structures pooled together). *Right*: Pairwise comparison between epi- and exocuticle for each parameter performed by t-test.

Parameter	EP, epicuticle			EX, exocuticle			t-test		
	Mean	SD	N	Mean	SD	N	t-ratio	DF	*p*-value
Ae	17.89	10.33	92	11.89	4.73	92	− 5.17204	120.6157	< .0001*
Ca	5.67	2.82	92	4.96	2.48	92	− 1.80084	179.1505	0.0367*
Cl	1.06	0.61	92	0.96	0.55	92	− 1.08347	180.4821	0.1400
F	1.87	1.07	92	1.46	0.94	92	− 2.72288	179.1478	0.0036*
Fe	0.39	0.27	92	0.35	0.22	92	− 1.17512	176.0772	0.1208
H	3.73	1.29	80	2.53	0.47	80	− 7.81806	99.58759	< .0001*
K	0.12	0.07	92	0.10	0.07	92	− 1.17248	181.097	0.1213
Mg	0.41	0.24	92	0.40	0.21	92	− 0.1011	178.8505	0.4598
Na	0.01	0.05	92	0.00	0.00	92	− 1.87772	92.65782	0.0318*
P	2.72	1.50	92	2.24	1.41	92	− 0.57312	174.8972	0.0159*
S	0.19	0.11	92	0.19	0.11	92	− 0.05926	181.4957	0.4764
Si	4.27	6.27	92	0.01	0.02	92	− 6.52317	91.00142	< .0001*
E	43.13	14.80	80	28.77	5.43	80	− 7.9688	95.49593	< .0001*
Zn	0.28	0.26	92	0.28	0.22	92	− 0.10207	176.4232	0.4594

*E* Young’s modulus, *EP* epicuticle, *EX* exocuticle, *H* hardness, *N* quantity of test, *SD* standard deviation.

When sorting the H and E values to the different localities tested, the highest values were measured for the (epicuticle of the) medial tooth—at its projections, plates, and the lateral part of the stylus; and for the (epicuticle of the) lateral tooth—at its posterior ridges (Fig. 6B and Supplementary Fig. 13). From these localities on, the structures became gradually softer and more flexible. The exocuticle underneath was softer and more flexible in all localities, but it was not homogeneous (Fig. 6D and Supplementary Fig. 13). The posterior process and the lateral edge of the medial tooth again showed the highest H and E values, together with the posterior ridges of the lateral tooth. Gradients, which could be determined for the epicuticle, were also pronounced in the exocuticle.

### Elemental composition determined by EDX

**Figure 7:**
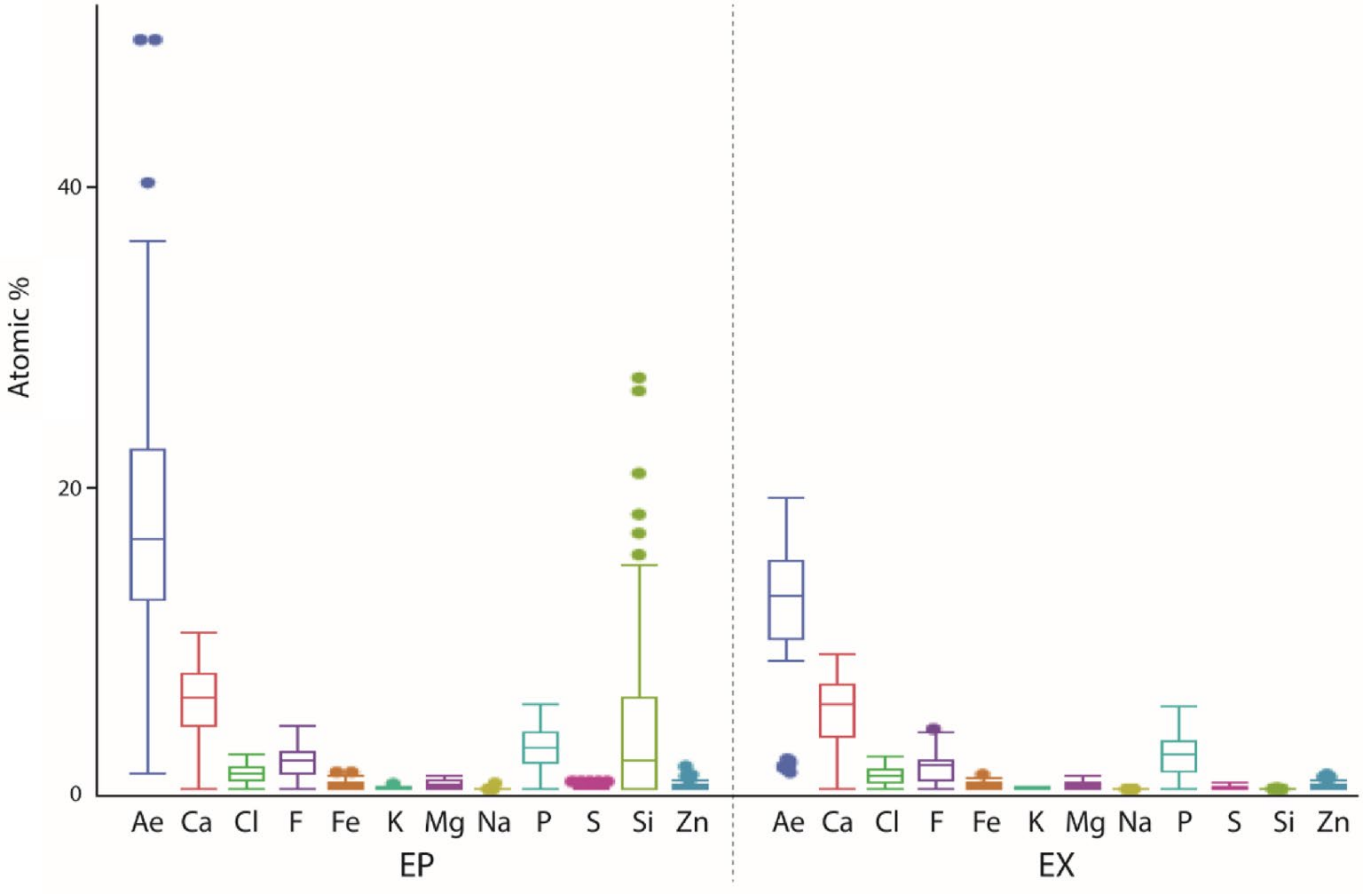
The results of elemental analysis (EDX) of the epicuticle and exocuticle (all structures studied are pooled together) (for exact values, see Table 2). The elemental proportions of Ae, Ca, Cl, F, Fe, K, Mg, Na, P, S, Si, and Zn are presented in atomic %. *Ae* all elements, *EP* epicuticle, *EX* exocuticle.

The elemental proportions (atomic ratio, atomic %) of Ca, Cl, F, Fe, K, Mg, Na, P, S, Si, and Zn were measured and analyzed (summarized to “all elements, Ae”). With regard to Ae, F, and Si, we detected highly significant differences and for Ca and Na significant differences between the epi- and the exocuticle (values from all structures pooled together) (see Table 2). Proportions of Cl, Fe, K, Mg, P, S, and Zn did not differ significantly between epi- and exocuticle. In general, we found higher proportions of elements in the epicuticle (see Figs. 6, 7 and Table 2): content of Ae, Ca, F, and Si was particularly higher and of Cl, Fe, K, Mg, Na, and P slightly higher in the epicuticle. S and Zn contents were similar in both epi- and exocuticle.

With regard to the individual structures analyzed (values of the epicuticle and exocuticle pooled together), we detected that most structures differed highly significantly in Ae, Ca, Cl, F, Fe, Mg, P, S, Si, and Zn content (Supplementary Table 2). Most structures did not show differences in K and Na proportions. For Ae (all elements) and many individual elements (Ca, Cl, P, Si), the teeth contained the highest proportions. However, for some elements (F, Fe, Mg, S) this was not the case, which makes the picture rather puzzling (please see Table 3, Supplementary Figs. 2, 3, 4, 5, Supplementary Table 1).

**Table 3:** Occurrence of all elements (Ae) and the individual elements in the studied structures. In each line, structures are ordered according to the proportions of the elements (*left side*: highest proportion, *right side*: smallest proportion).

Element	Highest proportions								Smallest proportions
Ae (all elements)	Medial tooth	Lateral tooth	Accessory tooth	Cardiopyloric valve	Pterocardiac ossicle	Lateral cardiac ossicle	Zygocardiac ossicle	Membrane	Setae
Ca	Lateral tooth	Medial tooth; accessory tooth	Pterocardiac ossicle	Zygocardiac ossicle	Cardiopyloric valve	Lateral cardiac ossicle	Setae	Membrane	
Cl	Lateral tooth	Medial tooth	Zygocardiac ossicle	Accessory tooth	Pterocardiac ossicle	Lateral cardiac ossicle	Cardiopyloric valve	Membrane	Not detected: setae
F	Cardiopyloric valve	Medial tooth	Lateral cardiac ossicle	Lateral tooth	Accessory tooth	Zygocardiac ossicle	Pterocardiac ossicle	Setae	Membrane
Fe (in traces)	Medial tooth	Zygocardiac ossicle	Cardiopyloric valve	Accessory tooth	Lateral tooth	Pterocardiac ossicle	Lateral cardiac ossicle	Not detected: setae; membrane	
K (in traces)	Accessory tooth	Cardiopyloric valve; lateral tooth; medial tooth; pterocardiac ossicle	Lateral cardiac ossicle; zygocardiac ossicle	Membrane	Setae				
Mg (in traces)	Cardiopyloric valve	Lateral cardiac ossicle	Pterocardiac ossicle	Accessory tooth	Medial tooth	Zygocardiac ossicle; lateral tooth	Membrane	Setae	
Na (in traces)	Accessory tooth	Pterocardiac ossicle	Zygocardiac ossicle	Not detected: cardiopyloric valve; lateral tooth; medial tooth; membrane; setae; lateral cardiac ossicle					
P	Accessory tooth	Lateral tooth	Pterocardiac ossicle	Cardiopyloric valve	Zygocardiac ossicle	Medial tooth	Lateral cardiac ossicle	Membrane (in traces)	Setae (in traces)
S (in traces)	Cardiopyloric valve	Lateral cardiac ossicle	Pterocardiac ossicle	Accessory tooth	Medial tooth; zygocardiac ossicle	Membrane	Lateral tooth	Setae	
Si	Medial tooth	Lateral tooth	Accessory tooth	Zygocardiac ossicle (in traces); pterocardiac ossicle (in traces)	Not detected: cardiopyloric valve; membrane; setae; lateral cardiac ossicle				
Zn (in traces)	Medial tooth	Cardiopyloric valve	Lateral cardiac ossicle	Zygocardiac ossicle; pterocardiac ossicle	Accessory tooth	Lateral tooth	Not detected: membrane; setae		

When sorting the data on the elemental proportions to the structures and cuticle layer (Table 1 and Supplementary Figs. 4, 5), the epicuticle contained more elements than the exocuticle in most cases. In the membrane however, contents of almost all elements were similar in both epi- and exocuticle. Thus, in most structures the epi- and the exocuticle we found to be distinct in material properties, whereas the membrane seemed to be more homogeneous with regard to the elemental distribution.

When sorting the elemental proportions to the different localities (Supplementary Figs. 7, 8, 9, 10, 11, 12), the highest values of Ae were determined (similar to the H and E values) for the (epicuticle of the) medial tooth—at its projections, plates, and the lateral part of the stylus; and for the (epicuticle of the) lateral tooth—at its posterior ridges and anterior cusps (Fig. 6A). From these localities on, the structures became less mineralized. The exocuticle underneath was less mineralized in all localities, but not completely homogeneous (Fig. 6C). The posterior process and the lateral edges of the medial tooth again possessed the highest elemental proportions. Gradients, which could be determined for the epicuticle, could be found in the exocuticle as well, but were not as pronounced. Si was found in high proportions in the (epicuticle of the) medial tooth—at its projections and plates; in the (epicuticle of the) lateral tooth—at its cusps and ridges; and in the (epicuticle of the) accessory tooth—at its tip. In general, Si incorporations were determined at the surfaces of the teeth which interact with the food. In the setae, the tips contained always more Ae and Ca than the bases (Supplementary Fig. 12).

### Relationship between parameters

Most parameters correlated positively (see Supplementary Tables 3, 4, 5, 6). E and H showed a very high positive correlation (r = 0.99); Ae and Ca, Ae and H, Ae and P, Ae and Si, Ae and E, Ca and Cl, Ca and A, H and Si, Si and E – high positive correlations (r = 0.75–0.87). Ae and Cl, Ae and Cl, Ae and F, Ae and Fe, Ae and Zn, Ca and F, Ca and Fe, Ca and Zn, Cl and F, Cl and P, F and Mg, F and P, F and Zn, Fe and Zn, K and P showed moderate positive correlations (r = 0.50–0.69). All other parameter pairs showed low or negligible correlations.

**Figure 8:**
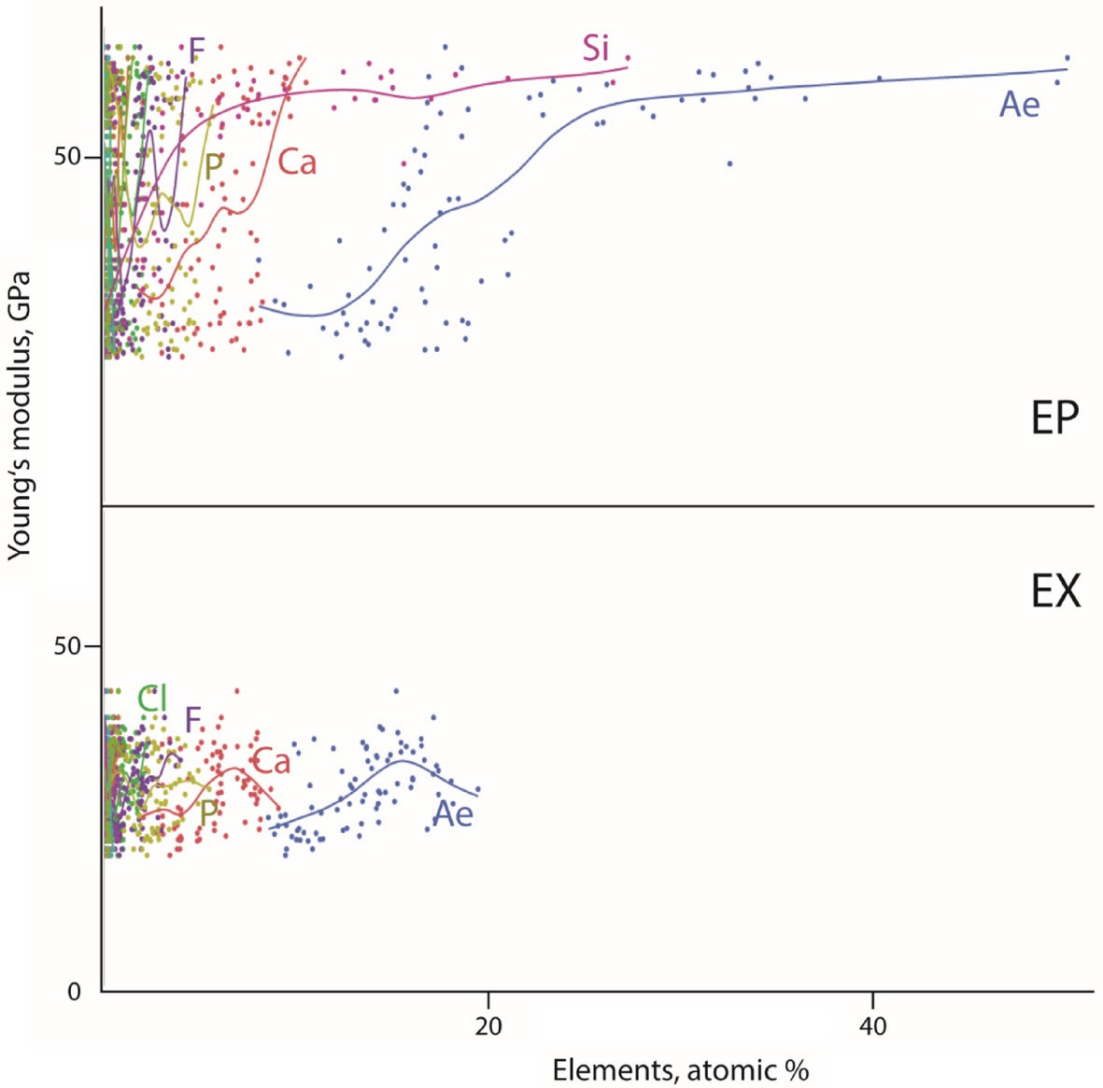
Relationship between the Young’s modulus, given in GPa, and the proportions, given in atomic %, of each individual element and Ae for the epicuticle (*above*) and the exocuticle (*below*). *Ae* all elements, *EP* epicuticle, *EX* exocuticle.

When plotting the Young’s modulus against the proportion of elements, the positive relationship becomes visible (between E and Ae, E and Si, E and Ca, E and F, E and P) (Supplementary Fig. 6). When, however, these values are sorted to either epi- or to exocuticle, we could detect that this relationship is especially pronounced for the epicuticle, whereas we could not determine such a relationship for the exocuticle (Fig. 8).

## Discussion

### Composition of the crustacean cuticle and the gastric mill

The crustacean epicuticle seems to be distinct between taxa [for a comprehensive review, see^65^]. It lacks chitin fibers and was found to be composed primarily of waxes with some clusters of calcium salts [for carapace, see^66–68^; for mandibles, see^69^]. In some previous studies, however, high Si^70–74^ and Ca^75^ content was detected in the mandibles. For the gastric mill, only Si, abundant with high proportions, was previously determined in the teeth^15,16^, which was detected here for the epicuticle of the teeth (5–18 atomic %). Our values are within the Si ranges, determined by previous studies on the mandibles of Copepoda (up to 29% Si^72,74^). This illustrates, that the epicuticle seems to be highly adapted to the environment and its specific function^69^, e.g. forming an osmiophilic waxy layer, which reduces water loss in terrestrial species^76,77^, or a thick layer in the mandibles or gastric mill teeth, which support the cutting and crushing of food^78,79^.

The exocuticle of Crustacea was previously found to contain stacked layers of chitin-protein fibrils with varying orientation (Bouligand or twisted plywood structures) [for carapace, see^67^; for extremities, see^53,55,56,80–83^; for carapace and extremities, see^50^]. This specific arrangement rendered the exocuticle a lightweight material of immensely high strength and toughness with crack deflection and reorientation [for a comprehensive review on toughening mechanisms, see^84^]. Here, a similar micro-structure was observed for the exocuticle of the gastric mill teeth and ossicles.

Previous studies determined, that calcium carbonate and calcium phosphate are located between the fibers of the crustacean exocuticle, reinforcing the structures and increasing chemical resistance [for gastric mill, see^15^; for carapace, see^67,68,85^; for carapace and extremities, see^50,52,86^; for mandibles, see^69,72,73,75,78,87^; for extremities, see^81,88^]. Cl was also previously detected [in the gastric mill, see^16^; in mandibles, see^72,73^]. Here, we identified Ca, P, Cl, and F in both epi- and exocuticle, which suggests, that potentially apatite (chlorine- and fluorine-apatite) is incorporated in the gastric mill of *Procambarus clarkii*. Apatite was previously also identified for the gastric mill teeth of the same species^13^ and in the cuticle of other crustaceans^75,81,83,89,90^.

Besides of Ca, Cl, and P, the following elements, that are not the basis of chitin and probably reinforce the cuticle, were previously detected in the cuticle of crustacean: Mg^50,51,55,69,78,81,88^; S, K, Br, Zn, Cu, Fe, and Ni in copepod mandibles^72^; Cu and Zn in copepod mandibles^71^; Br in amphipod mandibles^73^ and isopod claws^88^; S in shrimp claws^52^. For *Procambarus clarkii*, we also detected Mg, K, S, Zn, and Fe, but no Br, Ni, and Cu. Additionally, we detected Na, which, to the best of our knowledge, was not found in crustacean cuticle before.

For limpet radular teeth, which are also composed of chitin fibers, Mg and Ca were previously found to be involved in the protein packing, which is related to an increase in chitin fibre density of and in material stiffness^91^. Potentially, Mg and Ca are here also involved in stiffening of the chitin in crustaceans, but this, however, awaits further investigations. It was previously determined, that Na, K, and S can be related to the protein bonding and degree of tanning [e.g.,^92–94^] —potentially this is also the case in crustaceans. The detected proportions of Fe and Zn potentially also increase the stiffness and hardness in the gastric mill, comparable to molluscan teeth—even though, the detected proportions are very small in comparison to the incorporations in Polyplacophora and limpets [e.g.,^58,60,95,96^]. But this again awaits further investigations.

The hardness (H) is the measure of the resistance to local plastic deformation induced by indentation or abrasion. The Young’s modulus (E) indicates the stiffness of a solid material and describes the relationship between tensile stress and axial strain. It correlates with the ability of the material to transmit force, which is important to understand the puncturing behavior and failure resistance [for comprehensive review on puncture mechanics, see^64^].

With regard to the mechanical properties, previous studies on various crustacean taxa identified the following Young’s moduli: for dactyls, values range from 25 to 60 GPa^83^ and from 10 to 70 GPa^81^; for claws, from 2 to 26 GPa^52^ and from 5 to 32 GPa^88^; for mandibles, from 4 to 33 GPa^69^ and from 10 to 100 GPa^75^; for chelae, from 2 to 55 GPa^51^; for the exocuticle of carapaces, from 8 to 24 GPa^56^ and from 8 to 69 GPa^50^. Most of these studies showed that the values decrease from the interacting surface towards interior, which could also be seen for the gastric mill of *Procambarus clarkii*. The here received E and H values, especially of the masticatory surfaces’ epicuticle (i.e., plates, projections, cusps, ridges) from the medial and lateral teeth are high, however, within the range measured for dactyls, mandibles, chela, and carapaces.

### Origins of the gradients of mechanical properties

Functional gradients and heterogeneities can have their origin in the geometry, composition, and structure [for reviews, see^36,84^]. In crustacean exoskeletons, the chemical composition (i.e., degree of mineralization^75^), the dimension of the fibrous layers (i.e., increasing layer thickness from exo- to endocuticle^53,80,86^), the orientation of fibrils (i.e., the periodically helical arrangement^54,80,86^), or a combination of chemical and structural gradients with graded interfaces^81,82,90^ was previously found to cause local heterogeneities, which determine the function of structure [for review, see^65^]. Additionally, the local thickness of the epicuticle probably contributes to the function, e.g. its resistance to abrasion, as well. A high variation in thickness was previously determined for the gastric mill structures of the spider crab (Brachyura, Decapoda)^97^ and should be on focus in future studies.

For the gastric mill of *Procambarus clarkii*, we determined, as it was also determined in previous studies on crustacean cuticle [e.g.,^50,51,55,56,65,83^], that the hardness and elasticity values relate to the mineral content. This relationship is especially prominent in the epicuticle; here the mechanical properties seem to have their origin in the proportions and distribution of the following inorganic elements: Si (potentially bounded as silica), Ca, P, and F (potentially present as apatite).

In the exocuticle however, the mechanical properties seem to have their origin in the degree of tanning, since the mineral content does not fully relate to the measured mechanical properties. In previous studies on arthropod cuticle [e.g.,^98–112^] the laser excitation via CLSM allowed the identification of regions with the following dominating material composition, according to the protocol of^49^: (a) sclerotized, stiff cuticle was associated with a red signal; (b) weakly-sclerotized chitin with a green signal. Sometimes, in combination with the elastic and flexible protein resilin [for review, see^101^], these areas appeared brown, yellow, or pink in overlay, as resilin produces a blue signal. (c) Blue signals were identified as regions containing high proportions of resilin, or related proteins. These studies were performed on wings^106^, foot attachment devices and legs^107–109^, mouthparts^38,110^, or genitals^111,112^. The proposed relationship between emitted autofluorescence signal, received by following the protocol^49^, and the mechanical properties was previously cross-validated by AFM-nanoindentation for the hair of foot attachment devices in a lady bird beetle^107^.

For crustaceans, this CLSM protocol was previously applied to copepod mandibles^113,114^, revealing regions of high sclerotization (red signal), high resilin content (blue signal), and Si (green signal)—but it was never cross-validated on crustaceans before. For the gastric mill components of *Procambarus clarkii*, we can here relate the autofluorescence signals with the elemental content and the mechanical properties: the strong green signal in the masticatory surfaces of the teeth seems to be related to the Si content, similar to copepod mandibles. These structures are the hardest and stiffest elements. The surrounding areas of blue autofluorescence are softer and more flexible, probably resulting from less sclerotized chitin with higher protein/resilin content. This region could potentially serve as a shock absorber when interacting with a hard obstacle, similar to the copepod gnathobases^113,114^.

In the setae, we could identify regional heterogeneities by CLSM and EDX. The basis exhibited a green signal and the tip however a red signal, which indicates that the basis is probably less sclerotized and thus softer than the tip – which however awaits a cross-validation via nanoindentation using AFM.

### Functionality of structures

In general, the masticatory surfaces of the gastric mill (i.e., the epicuticle of the tooth cusps) were determined as the hardest and stiffest regions here. This high hardness probably reduces abrasion and the high stiffness the probability of structural failure during crushing and shredding [see^9,11,12,26,45,114,116^]. The stiff and hard ossicles probably stabilize the stomach during these actions, whereas the cardiopyloric valves prevent the food particles from returning into the stomach from the guts^26,45^.

The setae from different stomach regions of *Procambarus clarkii* are distinct in morphology, similar to the previously described gastric mill of Majidae and Tanaidacea^45,117^. As proposed for different species, the cuspidate setae of *P. clarkii* are potentially capable of grasping particles [see^118–121^], whereas the plumodenticulate setae probably filter them [see^118,121^]. The pappose setae could function as chemoreceptors [see^118,121^], gap sealants [see^118^], mechanoreceptors [see^118^], or scrapers [see^122^]. As the setae bases in *P. clarkii* seem less sclerotized and thus softer and more flexible than the tip, the setae are probably capable of bending and moving under the stomach fluid flow.

## Supplementary Information

Supplementary Information.

## Data Availability

The data is available in the Supplementary files and the raw data is available from the corresponding author on reasonable request.
