# Supplementary material for: Mechanical properties, degree of sclerotisation and elemental composition of the gastric mill in the red swamp crayfish Procambarus clarkii (Decapoda, Crustacea)
Source: Sci Rep. 2022 Oct 23;12:17799. doi: 10.1038/s41598-022-22724-w (PMC9588795; doi:10.1038/s41598-022-22724-w)
Supplement: Supplementary file 1 — Supplementary Information. [file 41598_2022_22724_MOESM1_ESM.pdf]

Mechanical properties, degree of sclerotisation and elemental composition of the gastric mill in the red swamp crayfish *Procambarus clarkii* (Decapoda, Crustacea)

Wencke Krings[1,2,3*], Jan-Ole Brütt[1,2], Stanislav N. Gorb[3]

[1]Department of Behavioral Biology, Institute of Cell and Systems Biology of Animals, Universität Hamburg, Martin-Luther-King-Platz 3, 20146 Hamburg, Germany

[2]Department of Mammalogy and Paleoanthropology, Leibniz Institute for the Analysis of Biodiversity Change, Martin-Luther-King-Platz 3, 20146 Hamburg, Germany

[3]Department of Functional Morphology and Biomechanics, Zoologisches Institut, Christian-Albrechts-Universität zu Kiel, Am Botanischen Garten 9, 24118 Kiel, Germany

*corresponding author: wencke.krings@uni-hamburg.de

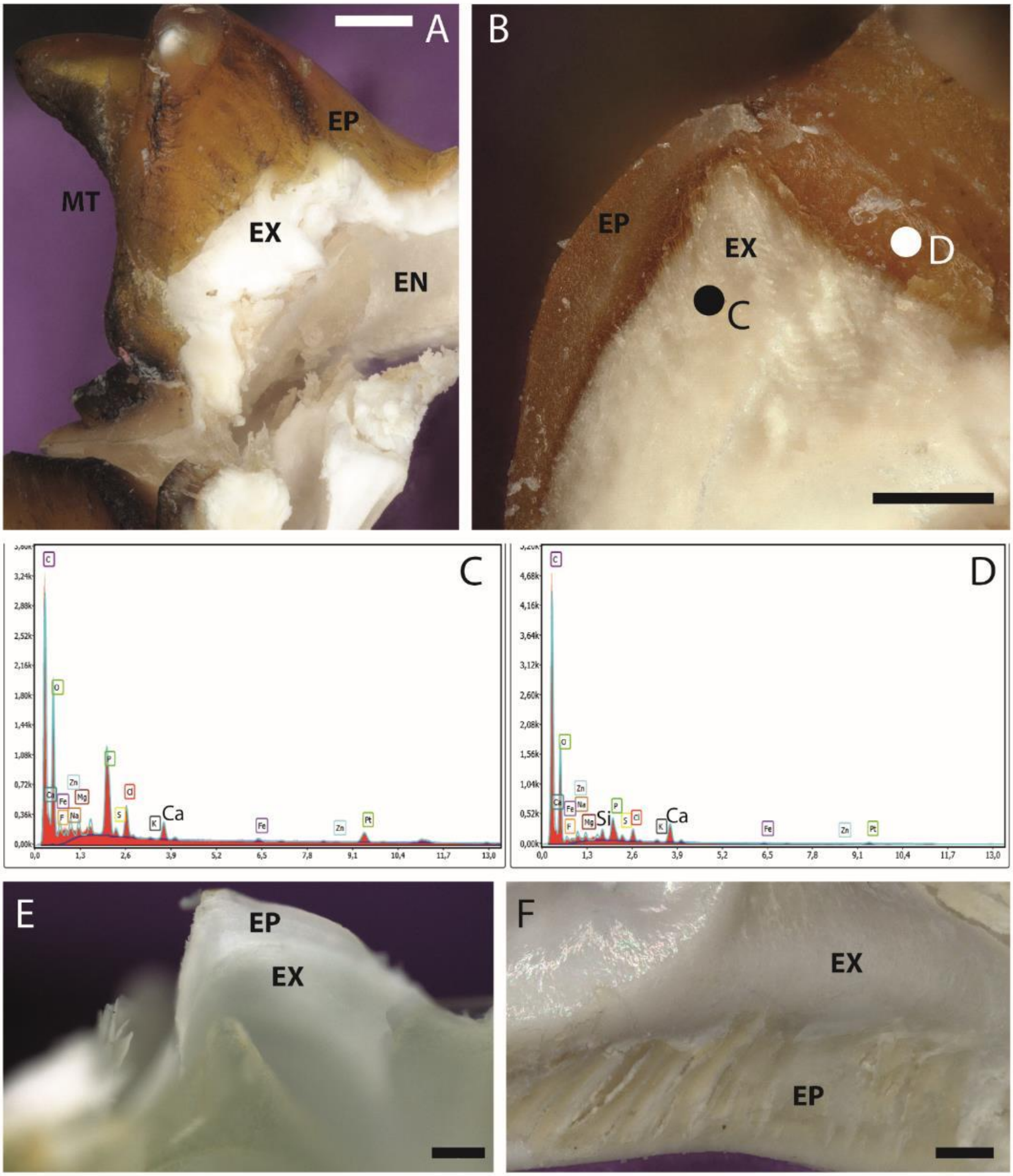

Supplementary Figure 1. A–B, E–F. Light microscopy images depicting the layered structure of the cuticula: epicuticle, exocuticle, and endocuticle. A. Medial tooth. B. Broken lateral tooth. C–D. EDX spectra of the exocuticle (C) and epicuticle (D). E. Broken pterocardiac ossicle. F. Broken zygocardiac ossicle. Abbreviations: EN, endocuticle; EP, epicuticle; EX, exocuticle; LT, lateral tooth; MT, medial tooth; PtO, pterocardiac ossicle; ZO, zygocardiac ossicle. Scale bars: A, 200 µm; B, 100 µm; E–F, 200 µm.

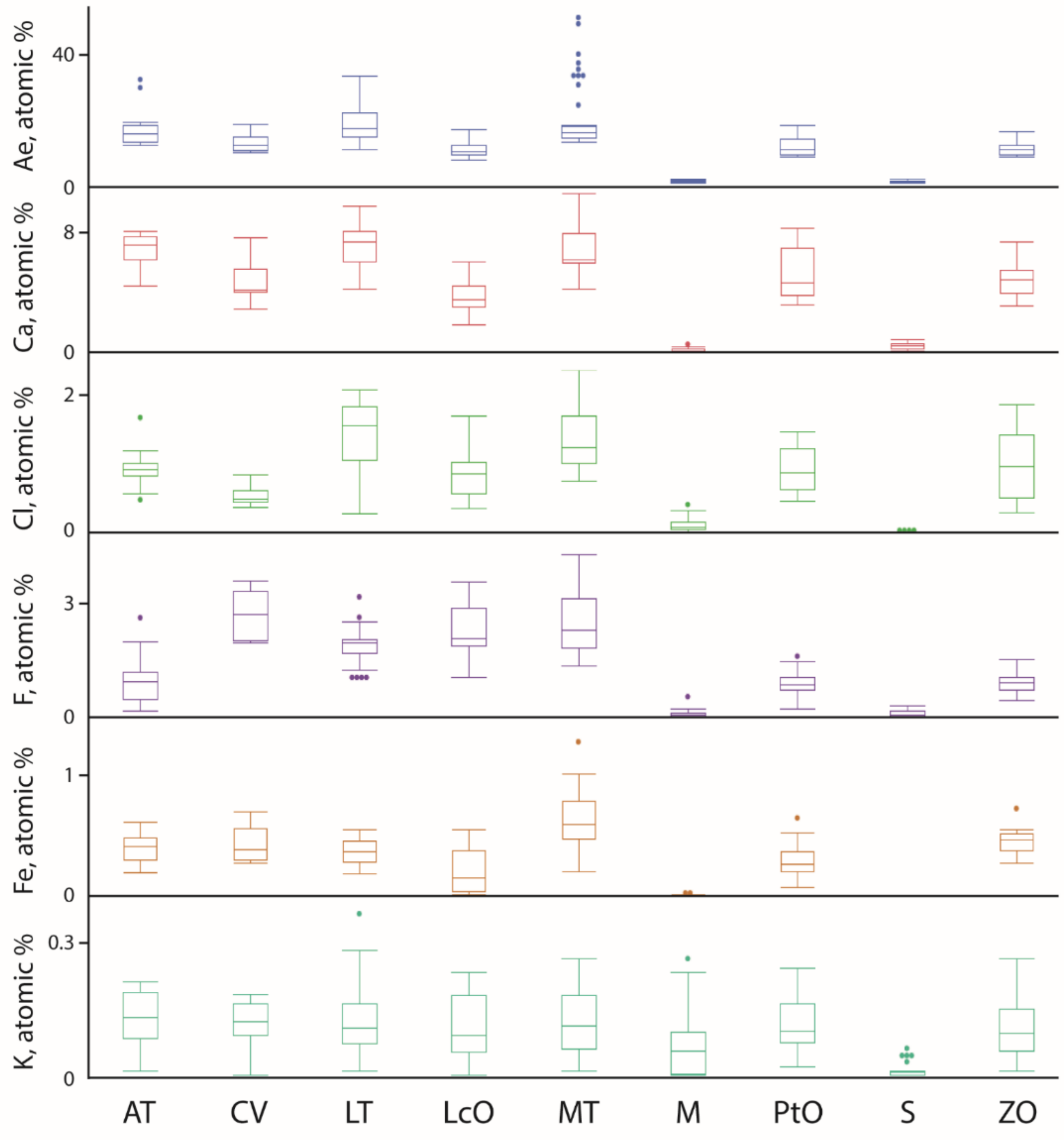

Supplementary Figure 2. The results of elemental analysis (EDX) of different structures tested (AT, CV, LcO, LT, MT, M, PtO, S, ZO) (for exact values, see Supplementary Table 2). The elemental proportions of Ae, Ca, Cl, F, Fe, and K are presented in atomic %. Abbreviations: Ae, all elements; AT, accessory tooth; CV, cardiopyloric valve; LcO, lateral cardiac ossicle; LT, lateral tooth; MT, medial tooth; M, membrane; PtO, pterocardiac ossicle; S, setae; ZO, zygocardiac ossicle.

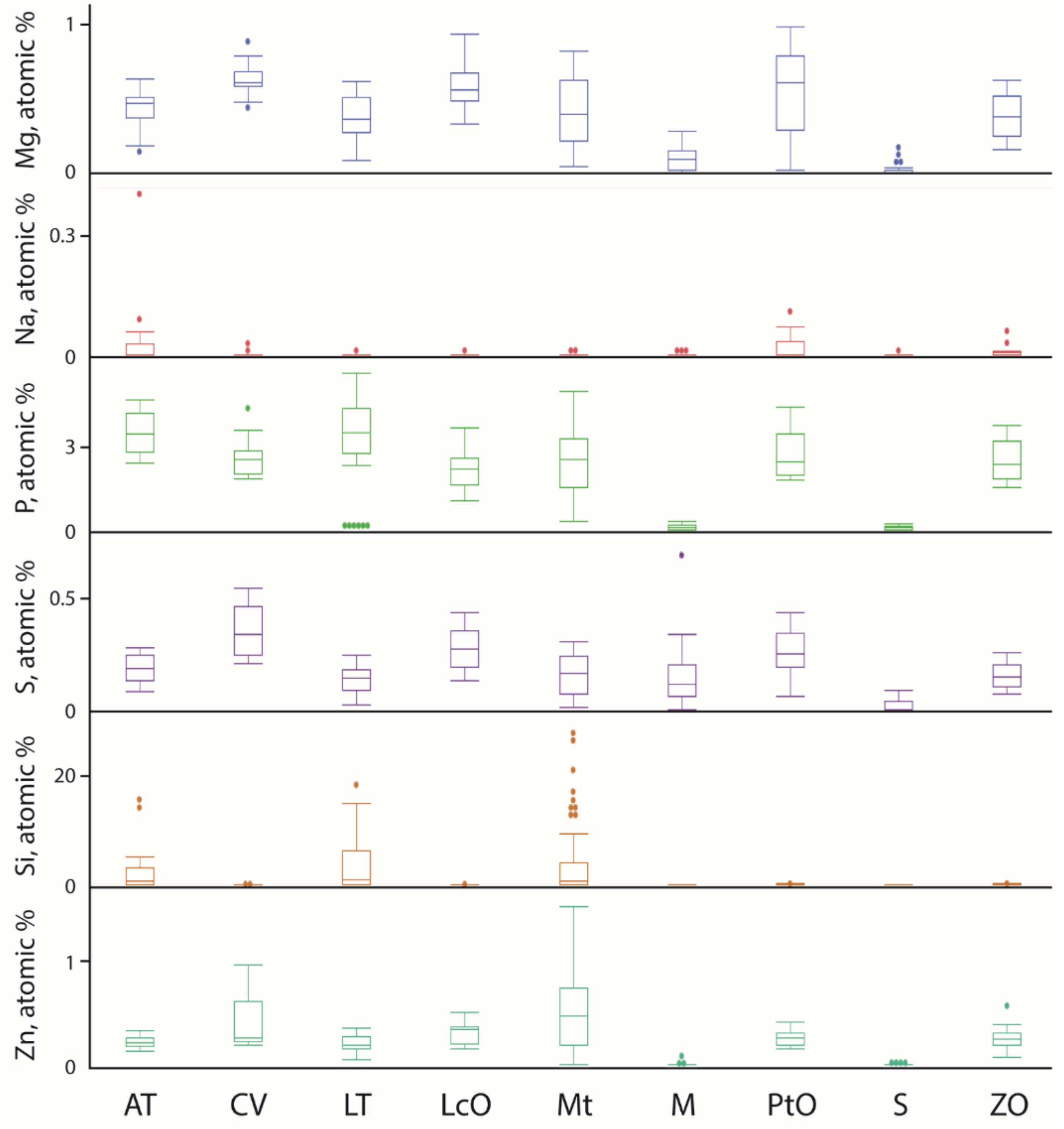

Supplementary Figure 3. The results of elemental analysis (EDX) of different structures tested (AT, CV, LcO, LT, MT, M, PtO, S, ZO) (for exact values, see Supplementary Table 2). The elemental proportions Mg, Na, P, S, Si, and Zn are presented in atomic %. Abbreviations: Ae, all elements; AT, accessory tooth; CV, cardiopyloric valve; LcO, lateral cardiac ossicle; LT, lateral tooth; MT, medial tooth; M, membrane; PtO, pterocardiac ossicle; S, setae; ZO, zygocardiac ossicle.

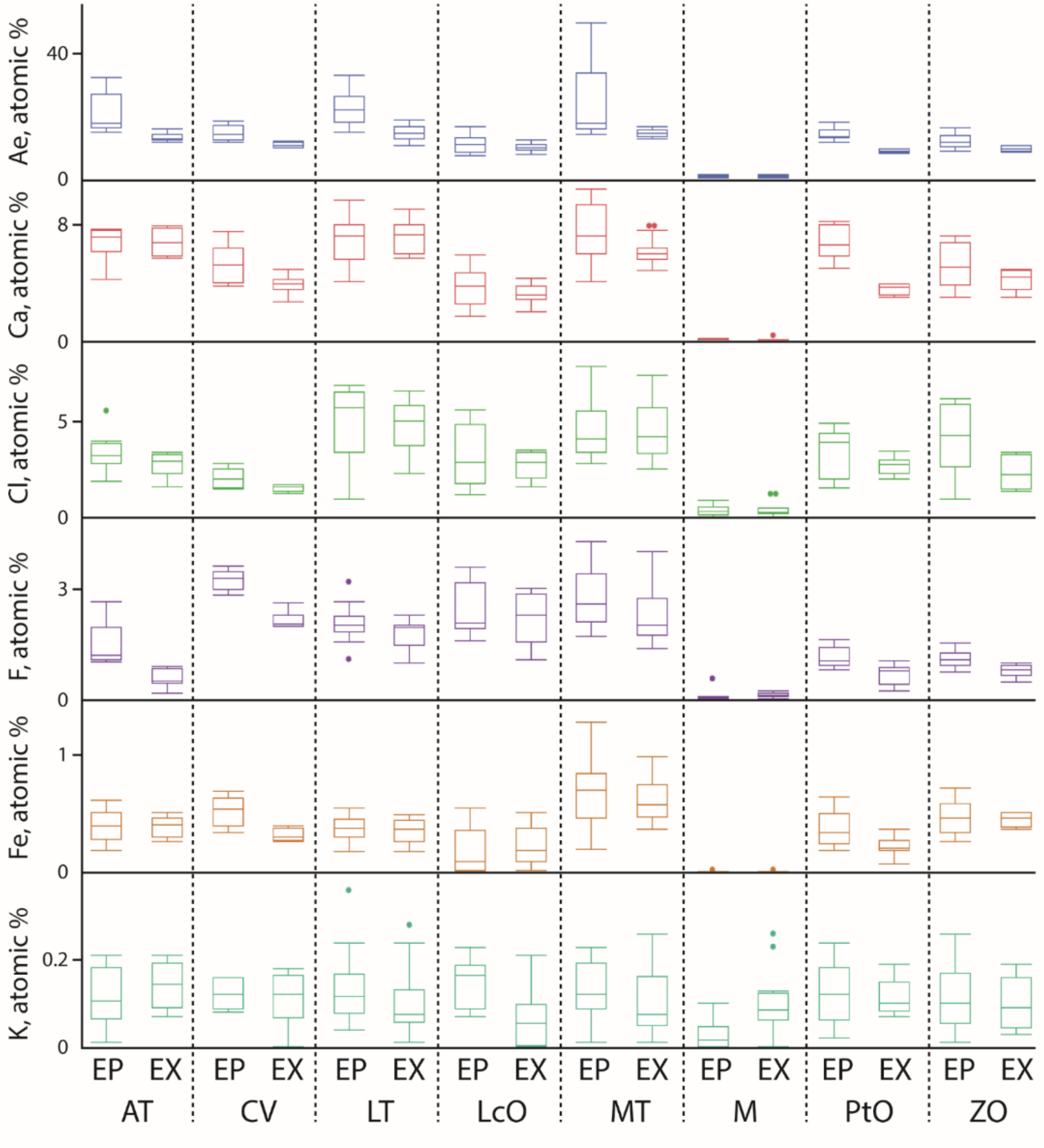

Supplementary Figure 4. The results of elemental analysis (EDX) of the epicuticle and exocuticle from different structures tested (AT, CV, LcO, LT, MT, M, PtO, S, ZO) (for exact values, see Table 2). The elemental proportions of Ae, Ca, Cl, F, Fe, and K are presented in atomic %. Abbreviations: Ae, all elements; AT, accessory tooth; CV, cardiopyloric valve; EP, epicuticle; EX, exocuticle; LcO, lateral cardiac ossicle; LT, lateral tooth; MT, medial tooth; M, membrane; PtO, pterocardiac ossicle; S, setae; ZO, zygocardiac ossicle.

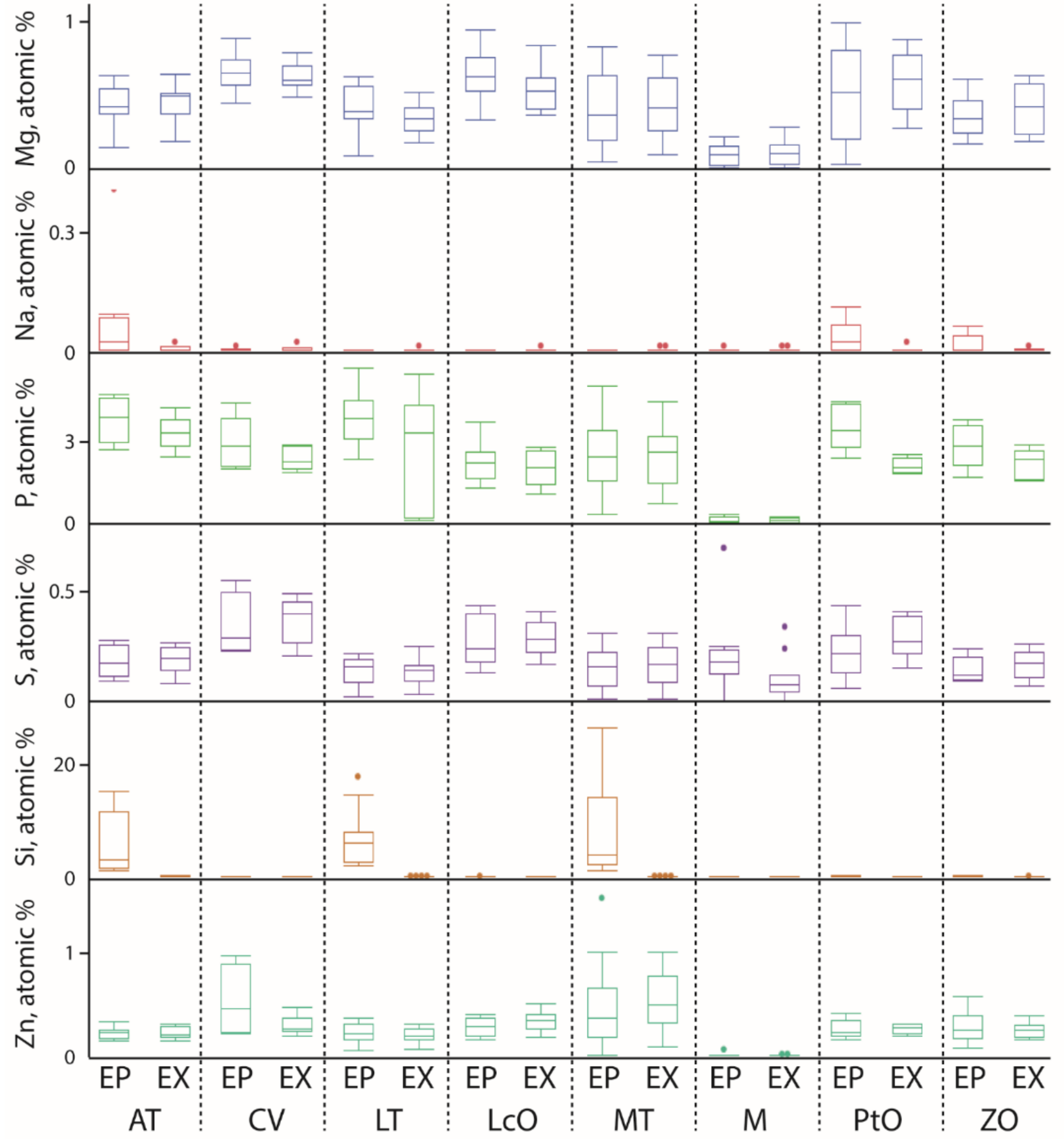

Supplementary Figure 5. The results of elemental analysis (EDX) of the epicuticle and exocuticle from different structures tested (AT, CV, LcO, LT, MT, M, PtO, S, ZO) (for exact values, see Table 2). The elemental proportions of Mg, Na, P, S, Si, and Zn are presented in atomic %. Abbreviations: AT, accessory tooth; CV, cardiopyloric valve; EP, epicuticle; EX, exocuticle; LcO, lateral cardiac ossicle; LT, lateral tooth; MT, medial tooth; M, membrane; PtO, pterocardiac ossicle; S, setae; ZO, zygocardiac ossicle.

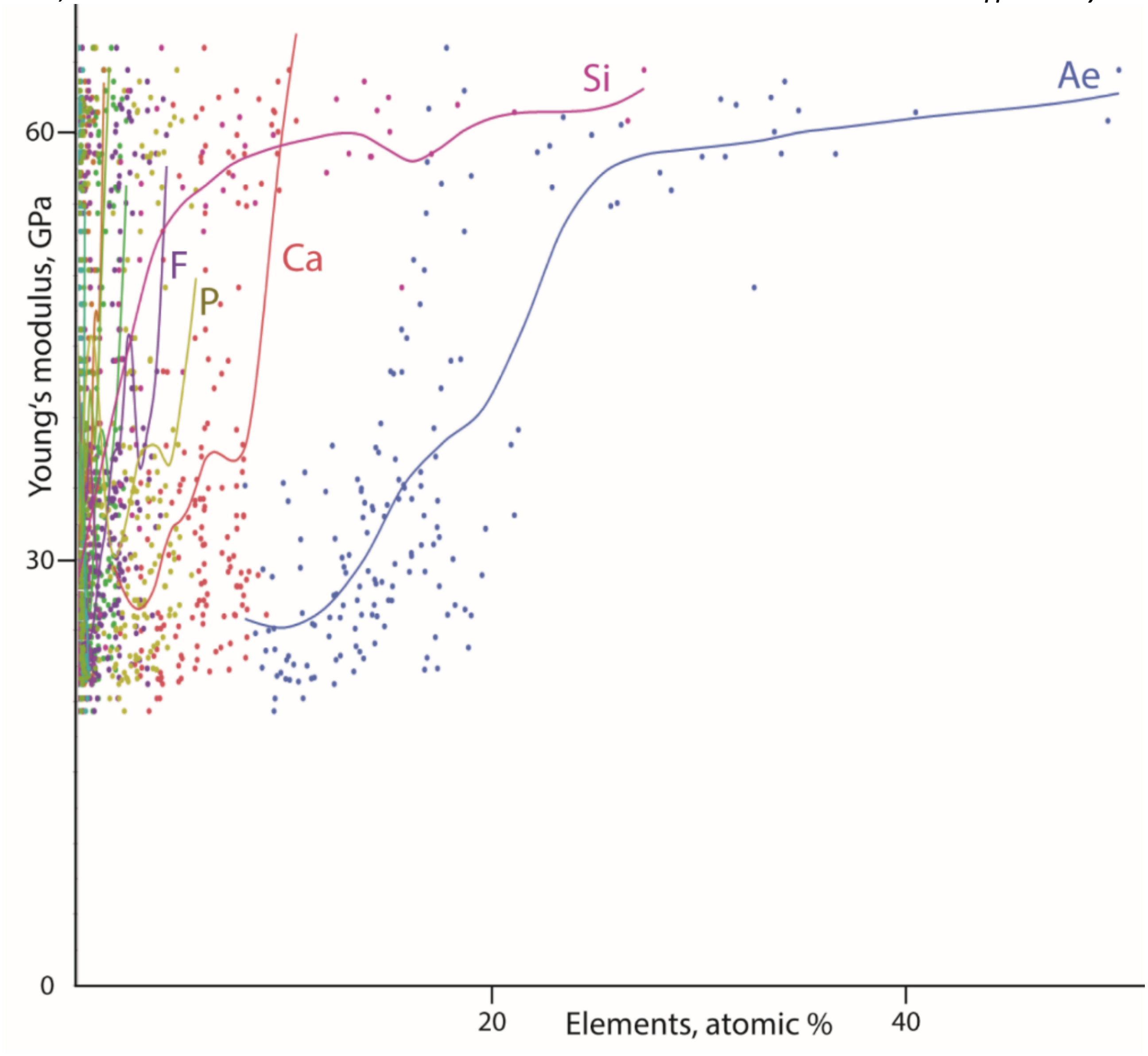

Supplementary Figure 6. Relationship between the Young's modulus (GPa), and the proportions (atomic %) of each individual element and all elements (Ae) in general (data from epi- and exocuticle and all structures pooled together). Abbreviations: Ae, all elements.

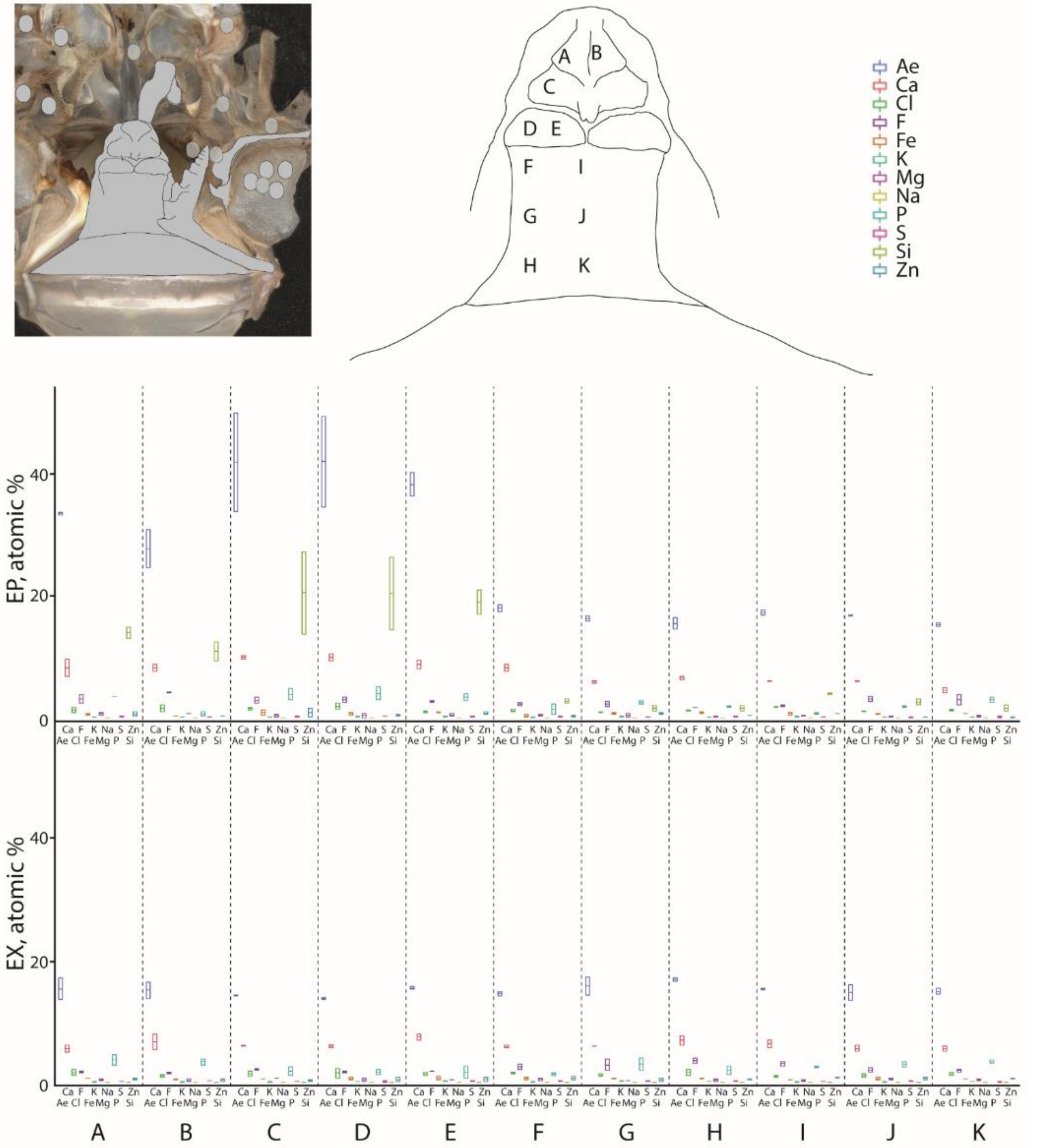

Supplementary Figure 7. The medial tooth: the EDX results in atomic % for the epi- and exocuticle (Ae, Ca, Cl, F, Fe, K, Mg, Na, P, S, Si, and Zn content) from the distinct localities tested (A–K). Each locality was tested in two individual stomachs. Abbreviations: Ae, all elements; EP, epicuticle; EX, exocuticle.

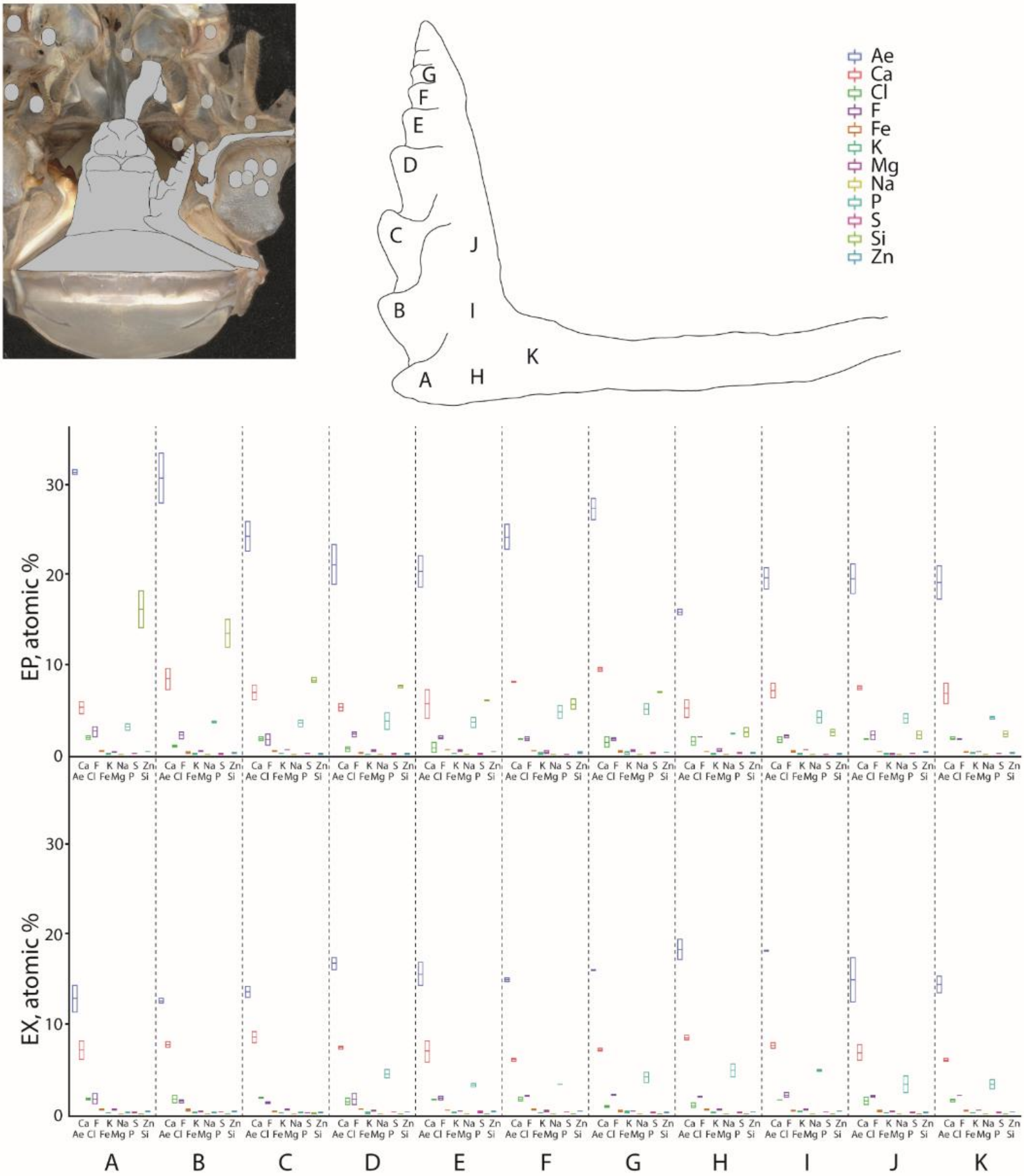

Supplementary Figure 8. The lateral tooth: the EDX results in atomic % for the epi- and exocuticle (Ae, Ca, Cl, F, Fe, K, Mg, Na, P, S, Si, and Zn content) from the distinct localities tested (A–K). Each locality was tested in two individual stomachs. Abbreviations: Ae, all elements; EP, epicuticle; EX, exocuticle.

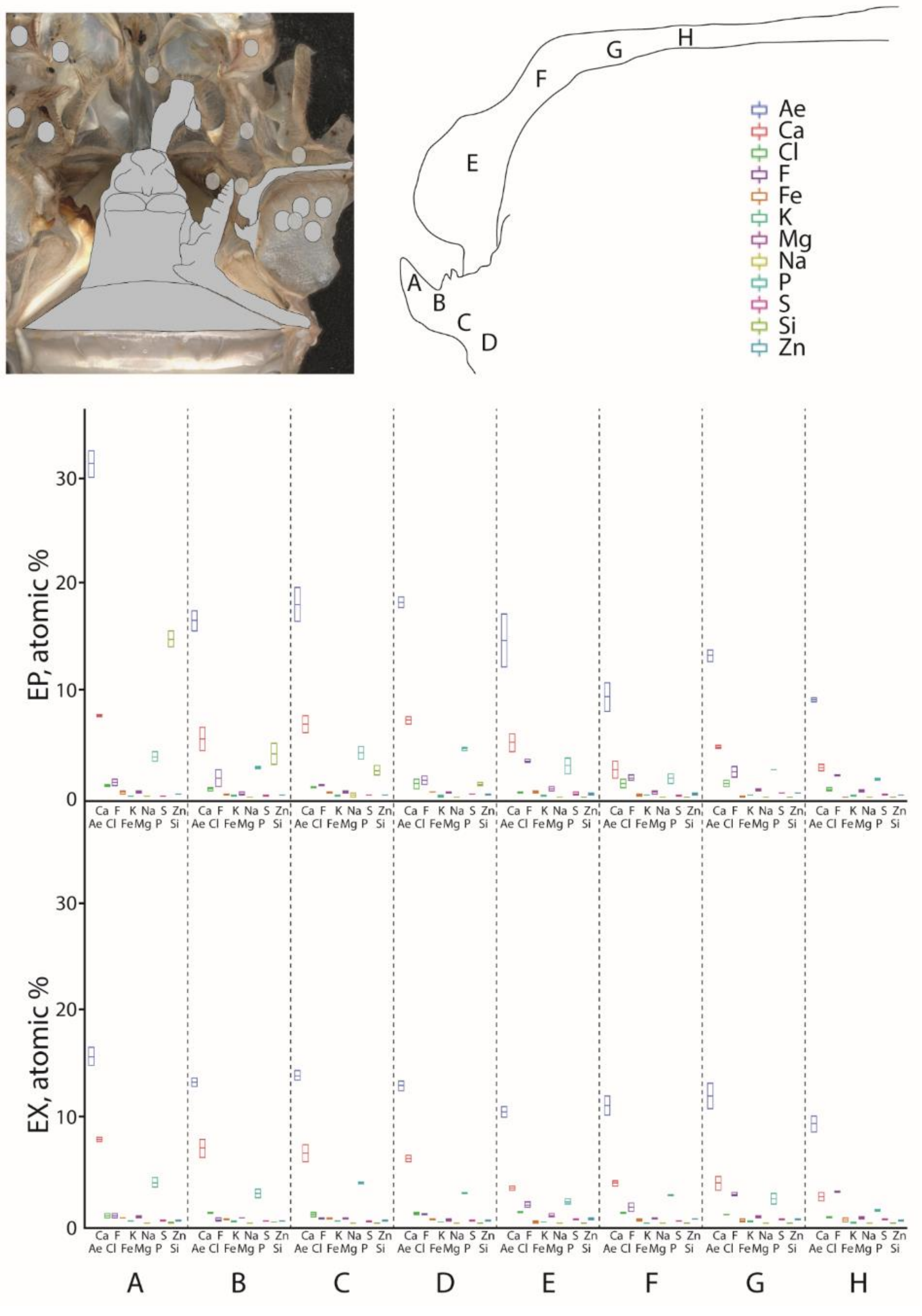

Supplementary Figure 9. The accessory tooth (A–D) and the lateral cardiac ossicle (E–H): the EDX results in atomic % for the epi- and exocuticle (Ae, Ca, Cl, F, Fe, K, Mg, Na, P, S, Si, and Zn content) from the distinct localities tested (A–H). Each locality was tested in two individual stomachs. Abbreviations: Ae, all elements; EP, epicuticle; EX, exocuticle.

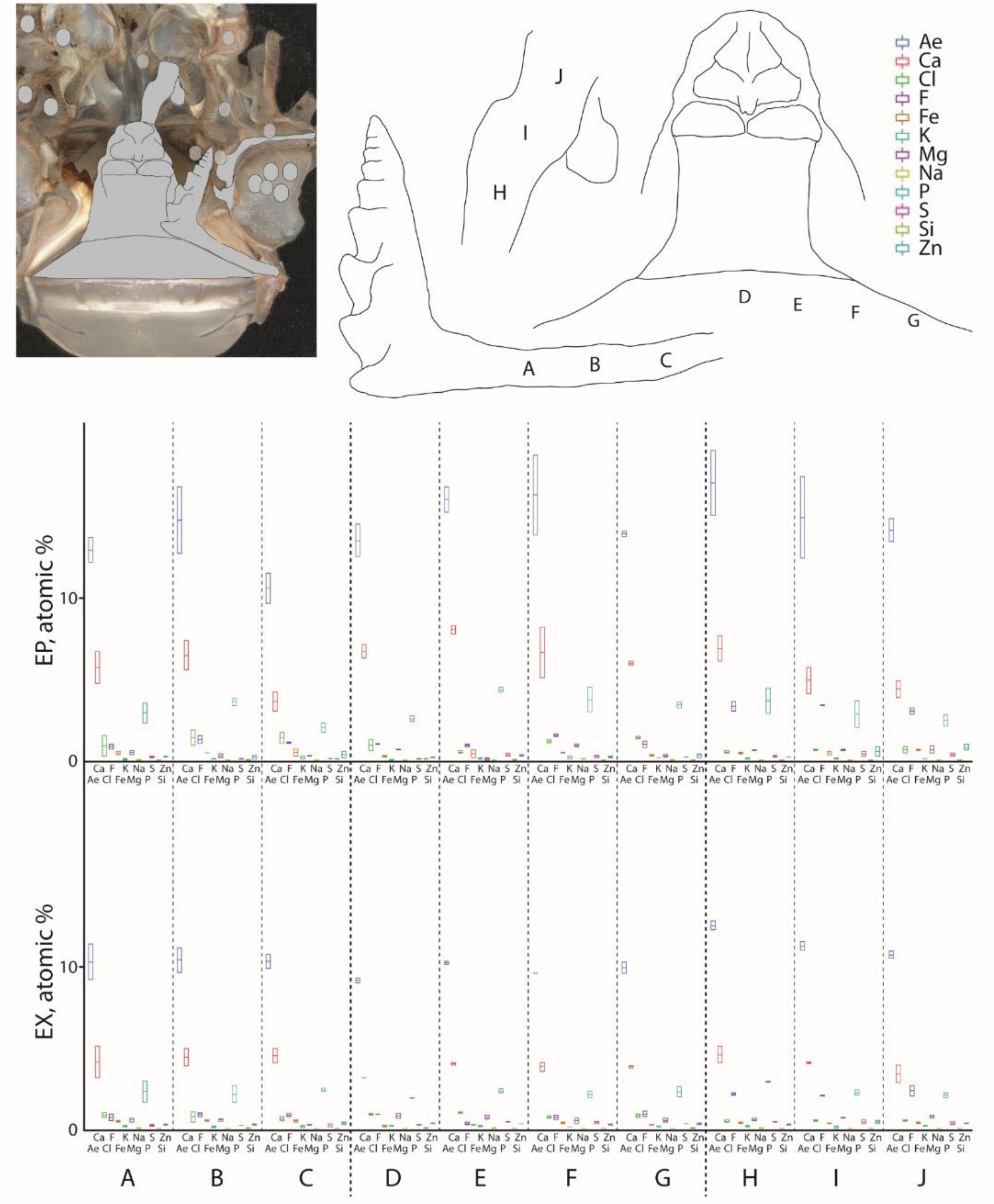

Supplementary Figure 10. The zygocardiac ossicle (A–C), the pterocardiac ossicle (D–G), and the cardiopyloric valve (H–J): the EDX results in atomic % for the epi- and exocuticle (Ae, Ca, Cl, F, Fe, K, Mg, Na, P, S, Si, and Zn content) from the distinct localities tested (A–J). Each locality was tested in two individual stomachs. Abbreviations: Ae, all elements; EP, epicuticle; EX, exocuticle.

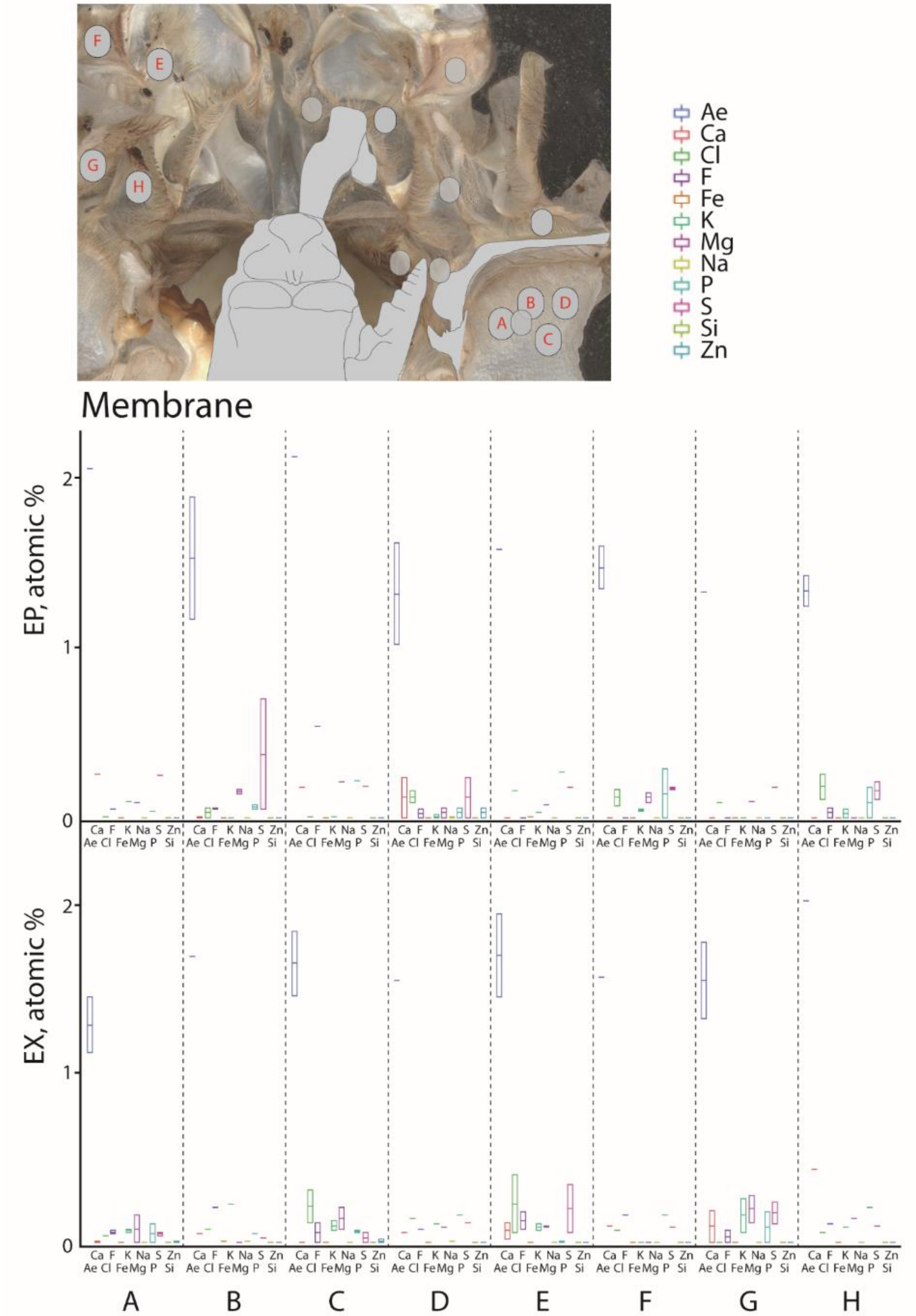

Supplementary Figure 11. The membrane: the EDX results in atomic % for the epi- and exocuticle (Ae, Ca, Cl, F, Fe, K, Mg, Na, P, S, Si, and Zn content) from the distinct localities tested (A–H). Each locality was tested in two individual stomachs. Abbreviations: Ae, all elements; EP, epicuticle; EX, exocuticle.

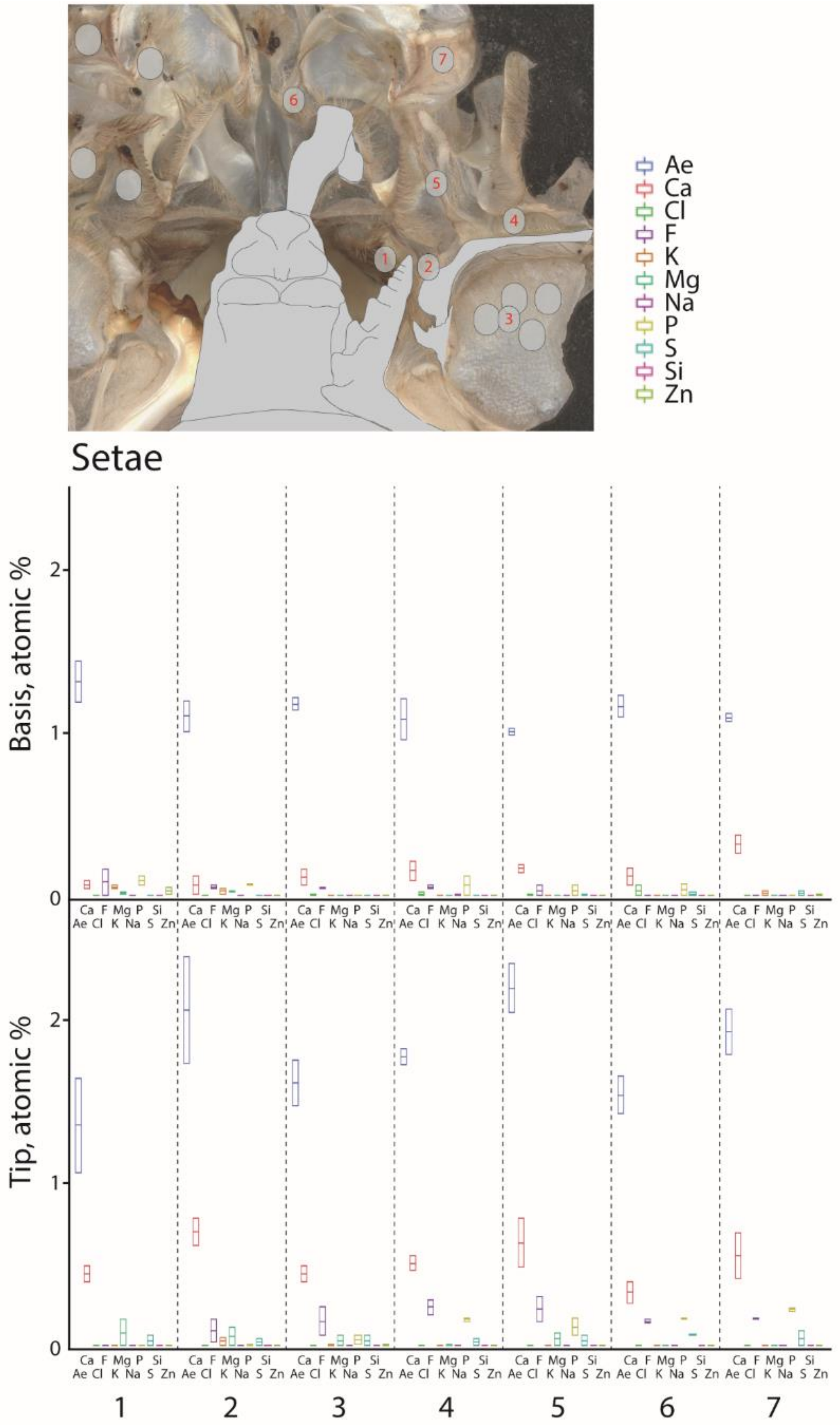

Supplementary Figure 12. The setae: the EDX results in atomic % (Ae, Ca, Cl, F, Fe, K, Mg, Na, P, S, Si, and Zn content) from the distinct localities of the tip and basis (1–7). Each locality was tested in two individual stomachs. Abbreviations: Ae, all elements.

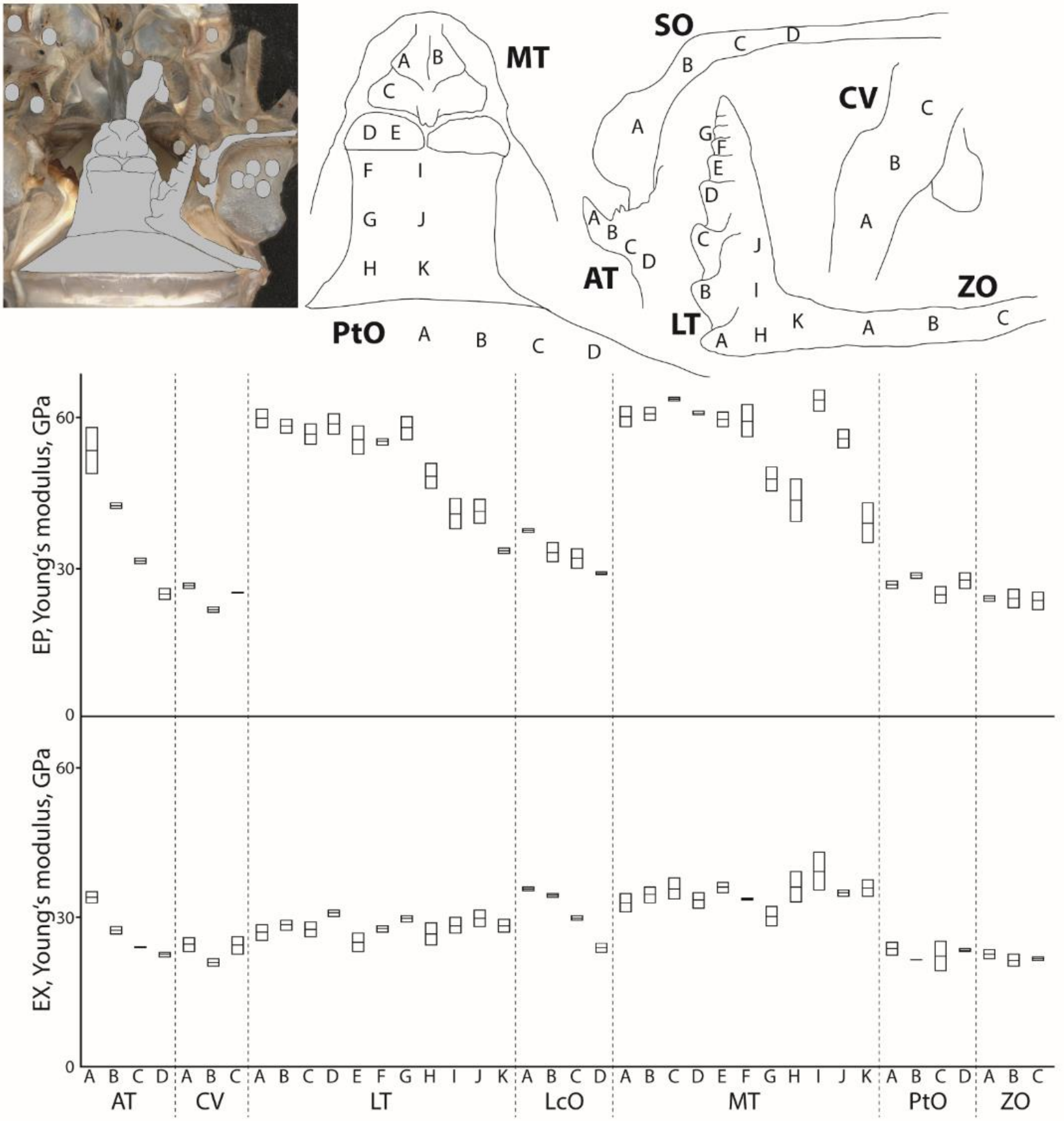

Supplementary Figure 13. The results of nanoindentation (Young's modulus, given in GPa) of the epi- and exocuticle from individual structures (AT, CV, LcO, LT, MT, PtO, ZO) at different localities. Abbreviations: AT, accessory tooth; CV, cardiopyloric valve; EP, epicuticle; EX, exocuticle; LcO, lateral cardiac ossicle; LT, lateral tooth; MT, medial tooth; PtO, pterocardiac ossicle; ZO, zygocardiac ossicle.

Supplementary Table 1. The mean and SD of the mechanical properties (H and E, both in GPa) and elemental proportions (in atomic %) for individual structures are listed. Abbreviations: E, Young's modulus; H, hardness; N, quantity of test; SD, standard deviation.

Structure		Ae	Ca	Cl	F	Fe	H	K	Mg	Na	P	S	Si	E	Zn
**AT, accessory tooth**	**Mean**	17,5	6,9	0,9	1,0	0,4	2,9	0,1	0,4	0,0	3,6	0,2	2,9	32,6	0,2
	**SD**	5,9	1,0	0,3	0,7	0,1	0,9	0,1	0,1	0,1	0,7	0,1	4,9	10,6	0,1
	**N**	16	16	16	16	16	16	16	16	16	16	16	16	16	16
**CV, cardiopyloric valve**	**Mean**	13,4	4,7	0,5	2,7	0,4	2,1	0,1	0,6	0,0	2,7	0,4	0,0	23,9	0,4
	**SD**	2,7	1,3	0,1	0,6	0,2	0,2	0,1	0,1	0,0	0,8	0,1	0,0	2,3	0,3
	**N**	12	12	12	12	12	12	12	12	12	12	12	12	12	12
**LT, lateral tooth**	**Mean**	19,2	7,1	1,5	1,9	0,4	3,4	0,1	0,4	0,0	3,4	0,1	3,4	40,0	0,2
	**SD**	5,6	1,4	0,5	0,4	0,1	1,0	0,1	0,1	0,0	1,5	0,1	4,7	13,7	0,1
	**N**	44	44	44	44	44	44	44	44	44	44	44	44	44	44
**MT, medial tooth**	**Mean**	20,5	6,9	1,3	2,5	0,6	4,0	0,1	0,4	0,0	2,5	0,2	4,5	45,5	0,5
	**SD**	9,8	1,6	0,4	0,8	0,2	1,2	0,1	0,2	0,0	1,2	0,1	7,5	12,6	0,3
	**N**	44	44	44	44	44	44	44	44	44	44	44	44	44	44
**M, membrane**	**Mean**	1,6	0,1	0,1	0,1	0,0	-	0,1	0,1	0,0	0,1	0,2	0,0	-	0,0
	**SD**	0,3	0,1	0,1	0,1	0,0	-	0,1	0,1	0,0	0,0	0,2	0,0	-	0,0
	**N**	24	24	24	24	24	-	24	24	24	24	24	24	-	24
**PtO, pterocardiac ossicle**	**Mean**	12,3	5,2	0,9	0,9	0,3	2,2	0,1	0,5	0,0	2,8	0,3	0,0	24,8	0,3
	**SD**	3,1	1,9	0,3	0,4	0,2	0,2	0,1	0,3	0,0	0,9	0,1	0,0	2,9	0,1
	**N**	16	16	16	16	16	16	16	16	16	16	16	16	16	16
**S, setae**	**Mean**	1,5	0,3	0,0	0,1	0,00	-	0,0	0,0	0,0	0,1	0,0	0,00	-	0,0
	**SD**	0,4	0,2	0,0	0,1	0,00	-	0,0	0,0	0,0	0,1	0,0	0,00	-	0,0
	**N**	28	28	28	28	28	28	28	28	28	28	28	28	-	28
**LcO, lateral cardiac ossicle**	**Mean**	11,2	3,6	0,9	2,3	0,2	2,8	0,1	0,6	0,0	2,1	0,3	0,0	32,0	0,3
	**SD**	2,3	1,1	0,4	0,7	0,2	0,4	0,1	0,2	0,0	0,7	0,1	0,0	4,4	0,1
	**N**	16	16	16	16	16	16	16	16	16	16	16	16	16	16
**ZO, zygocardiac ossicle**	**Mean**	11,5	4,8	1,0	0,9	0,5	2,0	0,1	0,4	0,0	2,5	0,2	0,0	22,9	0,3
	**SD**	2,2	1,3	0,5	0,3	0,1	0,2	0,1	0,2	0,0	0,8	0,1	0,0	1,7	0,1
	**N**	12	12	12	12	12	12	12	12	12	12	12	12	12	12

Supplementary Table 2. Chi-Square test followed by pairwise comparison, performed by Wilcoxon method, between the structures for each individual parameter (elements and mechanical properties). Orange p-values = highly significant, red = significant, black = not significant.

Element	Structure 1	Structure 2	1-Way Test, Chi-Square approximation			Wilcoxon Method, p-values
			Chi-Square	DF	p-values	
**Ae**	LcO, lateral cardiac ossicle	S, setae	160.2208	8	<.0001*	<.0001*
	PtO, pterocardiac ossicle	M, membrane				<.0001*
	LcO, lateral cardiac ossicle	M, membrane				<.0001*
	ZO, zygocardiac ossicle	S, setae				<.0001*
	ZO, zygocardiac ossicle	M, membrane				<.0001*
	MT, medial tooth	CV, cardiopyloric valve				0.0022*
	MT, medial tooth	AT, accessory tooth				0.0124*
	LT, lateral tooth	CV, cardiopyloric valve				0.0180*
	LT, lateral tooth	AT, accessory tooth				0.1085
	MT, medial tooth	LT, lateral tooth				0.1647
	CV, cardiopyloric valve	AT, accessory tooth				0.7628
	LcO, lateral cardiac ossicle	PtO, pterocardiac ossicle				0.7487
	ZO, zygocardiac ossicle	PtO, pterocardiac ossicle				0.5934
	ZO, zygocardiac ossicle	LcO, lateral cardiac ossicle				0.4861
	S, setae	M, membrane				0.1575
	PtO, pterocardiac ossicle	CV, cardiopyloric valve				0.0244*
	PtO, pterocardiac ossicle	AT, accessory tooth				0.0104*
	LcO, lateral cardiac ossicle	CV, cardiopyloric valve				0.0024*
	ZO, zygocardiac ossicle	CV, cardiopyloric valve				0.0009*
	LcO, lateral cardiac ossicle	AT, accessory tooth				0.0010*
	ZO, zygocardiac ossicle	AT, accessory tooth				0.0003*
	M, membrane	CV, cardiopyloric valve				<.0001*
	S, setae	CV, cardiopyloric valve				<.0001*
	M, membrane	AT, accessory tooth				<.0001*
	S, setae	AT, accessory tooth				<.0001*
	S, setae	PtO, pterocardiac ossicle				<.0001*
	PtO, pterocardiac ossicle	LT, lateral tooth				<.0001*
	ZO, zygocardiac ossicle	LT, lateral tooth				<.0001*
	LcO, lateral cardiac ossicle	LT, lateral tooth				<.0001*
	ZO, zygocardiac ossicle	MT, medial tooth				<.0001*
	PtO, pterocardiac ossicle	MT, medial tooth				<.0001*
	LcO, lateral cardiac ossicle	MT, medial tooth				<.0001*
	M, membrane	LT, lateral tooth				<.0001*
	M, membrane	MT, medial tooth				<.0001*
	S, setae	LT, lateral tooth				<.0001*
	S, setae	MT, medial tooth				<.0001*
**Ca**	LT, lateral tooth	CV, cardiopyloric valve	156.9970	8	<.0001*	<.0001*
	LcO, lateral cardiac ossicle	S, setae				<.0001*
	MT, medial tooth	CV, cardiopyloric valve				<.0001*
	PtO, pterocardiac ossicle	M, membrane				<.0001*
	LcO, lateral cardiac ossicle	M, membrane				<.0001*
	ZO, zygocardiac ossicle	S, setae				<.0001*
	S, setae	M, membrane				<.0001*
	ZO, zygocardiac ossicle	M, membrane				<.0001*
	ZO, zygocardiac ossicle	LcO, lateral cardiac ossicle				0.0178*
	LT, lateral tooth	AT, accessory tooth				0.5416
	PtO, pterocardiac ossicle	CV, cardiopyloric valve				0.7628
	ZO, zygocardiac ossicle	CV, cardiopyloric valve				0.8623
	ZO, zygocardiac ossicle	PtO, pterocardiac ossicle				0.6591
	MT, medial tooth	AT, accessory tooth				0.4773
	MT, medial tooth	LT, lateral tooth				0.2442
	LcO, lateral cardiac ossicle	CV, cardiopyloric valve				0.0327*
	PtO, pterocardiac ossicle	AT, accessory tooth				0.0194*
	LcO, lateral cardiac ossicle	PtO, pterocardiac ossicle				0.0066*
	ZO, zygocardiac ossicle	AT, accessory tooth				0.0005*
	CV, cardiopyloric valve	AT, accessory tooth				0.0003*
	PtO, pterocardiac ossicle	MT, medial tooth				0.0063*
	LcO, lateral cardiac ossicle	AT, accessory tooth				<.0001*
	PtO, pterocardiac ossicle	LT, lateral tooth				0.0016*
	M, membrane	CV, cardiopyloric valve				<.0001*
	S, setae	CV, cardiopyloric valve				<.0001*
	M, membrane	AT, accessory tooth				<.0001*
	ZO, zygocardiac ossicle	MT, medial tooth				0.0001*
	ZO, zygocardiac ossicle	LT, lateral tooth				<.0001*

	S, setae	AT, accessory tooth				<.0001*
	S, setae	PtO, pterocardiac ossicle				<.0001*
	LcO, lateral cardiac ossicle	LT, lateral tooth				<.0001*
	LcO, lateral cardiac ossicle	MT, medial tooth				<.0001*
	M, membrane	LT, lateral tooth				<.0001*
	M, membrane	MT, medial tooth				<.0001*
	S, setae	LT, lateral tooth				<.0001*
	S, setae	MT, medial tooth				<.0001*
**Cl**	MT, medial tooth	CV, cardiopyloric valve	153.2867	8	<.0001*	<.0001*
	LT, lateral tooth	CV, cardiopyloric valve				<.0001*
	LcO, lateral cardiac ossicle	S, setae				<.0001*
	PtO, pterocardiac ossicle	M, membrane				<.0001*
	ZO, zygocardiac ossicle	S, setae				<.0001*
	LcO, lateral cardiac ossicle	M, membrane				<.0001*
	LT, lateral tooth	AT, accessory tooth				0.0001*
	MT, medial tooth	AT, accessory tooth				0.0002*
	ZO, zygocardiac ossicle	M, membrane				<.0001*
	PtO, pterocardiac ossicle	CV, cardiopyloric valve				0.0005*
	LcO, lateral cardiac ossicle	CV, cardiopyloric valve				0.0050*
	ZO, zygocardiac ossicle	CV, cardiopyloric valve				0.0350*
	ZO, zygocardiac ossicle	LcO, lateral cardiac ossicle				0.7806
	ZO, zygocardiac ossicle	PtO, pterocardiac ossicle				0.9260
	ZO, zygocardiac ossicle	AT, accessory tooth				0.9445
	PtO, pterocardiac ossicle	AT, accessory tooth				0.8357
	LcO, lateral cardiac ossicle	PtO, pterocardiac ossicle				0.7486
	LcO, lateral cardiac ossicle	AT, accessory tooth				0.5214
	MT, medial tooth	LT, lateral tooth				0.1790
	CV, cardiopyloric valve	AT, accessory tooth				0.0002*
	ZO, zygocardiac ossicle	MT, medial tooth				0.0166*
	ZO, zygocardiac ossicle	LT, lateral tooth				0.0094*
	PtO, pterocardiac ossicle	MT, medial tooth				0.0006*
	M, membrane	CV, cardiopyloric valve				<.0001*
	LcO, lateral cardiac ossicle	MT, medial tooth				0.0002*
	LcO, lateral cardiac ossicle	LT, lateral tooth				0.0001*
	S, setae	CV, cardiopyloric valve				<.0001*
	M, membrane	AT, accessory tooth				<.0001*
	PtO, pterocardiac ossicle	LT, lateral tooth				<.0001*
	S, setae	M, membrane				<.0001*
	S, setae	AT, accessory tooth				<.0001*
	S, setae	PtO, pterocardiac ossicle				<.0001*
	M, membrane	LT, lateral tooth				<.0001*
	M, membrane	MT, medial tooth				<.0001*
	S, setae	LT, lateral tooth				<.0001*
	S, setae	MT, medial tooth				<.0001*
**F**	MT, medial tooth	AT, accessory tooth	167.9770	8	<.0001*	<.0001*
	LT, lateral tooth	AT, accessory tooth				<.0001*
	LcO, lateral cardiac ossicle	S, setae				<.0001*
	LcO, lateral cardiac ossicle	M, membrane				<.0001*
	ZO, zygocardiac ossicle	S, setae				<.0001*
	PtO, pterocardiac ossicle	M, membrane				<.0001*
	ZO, zygocardiac ossicle	M, membrane				<.0001*
	MT, medial tooth	LT, lateral tooth				0.0011*
	LcO, lateral cardiac ossicle	PtO, pterocardiac ossicle				<.0001*
	LcO, lateral cardiac ossicle	AT, accessory tooth				<.0001*
	CV, cardiopyloric valve	AT, accessory tooth				<.0001*
	LcO, lateral cardiac ossicle	LT, lateral tooth				0.0447*
	S, setae	M, membrane				0.6610
	ZO, zygocardiac ossicle	PtO, pterocardiac ossicle				0.8709
	ZO, zygocardiac ossicle	AT, accessory tooth				0.7983
	PtO, pterocardiac ossicle	AT, accessory tooth				0.6784
	LcO, lateral cardiac ossicle	MT, medial tooth				0.6337
	LcO, lateral cardiac ossicle	CV, cardiopyloric valve				0.1499
	MT, medial tooth	CV, cardiopyloric valve				0.2990
	ZO, zygocardiac ossicle	CV, cardiopyloric valve				<.0001*
	ZO, zygocardiac ossicle	LcO, lateral cardiac ossicle				<.0001*
	PtO, pterocardiac ossicle	CV, cardiopyloric valve				<.0001*
	M, membrane	CV, cardiopyloric valve				<.0001*
	LT, lateral tooth	CV, cardiopyloric valve				0.0005*
	M, membrane	AT, accessory tooth				<.0001*
	S, setae	CV, cardiopyloric valve				<.0001*
	S, setae	AT, accessory tooth				<.0001*

	S, setae	PtO, pterocardiac ossicle				<.0001*
	ZO, zygocardiac ossicle	LT, lateral tooth				<.0001*
	ZO, zygocardiac ossicle	MT, medial tooth				<.0001*
	PtO, pterocardiac ossicle	LT, lateral tooth				<.0001*
	PtO, pterocardiac ossicle	MT, medial tooth				<.0001*
	M, membrane	LT, lateral tooth				<.0001*
	M, membrane	MT, medial tooth				<.0001*
	S, setae	LT, lateral tooth				<.0001*
	S, setae	MT, medial tooth				<.0001*
**Fe**	MT, medial tooth	LT, lateral tooth	110.3975	7	<.0001*	<.0001*
	PtO, pterocardiac ossicle	M, membrane				<.0001*
	MT, medial tooth	AT, accessory tooth				0.0001*
	ZO, zygocardiac ossicle	M, membrane				<.0001*
	LcO, lateral cardiac ossicle	M, membrane				<.0001*
	MT, medial tooth	CV, cardiopyloric valve				0.0026*
	ZO, zygocardiac ossicle	LT, lateral tooth				0.0117*
	ZO, zygocardiac ossicle	LcO, lateral cardiac ossicle				0.0018*
	ZO, zygocardiac ossicle	PtO, pterocardiac ossicle				0.0036*
	ZO, zygocardiac ossicle	AT, accessory tooth				0.1698
	ZO, zygocardiac ossicle	CV, cardiopyloric valve				0.3547
	CV, cardiopyloric valve	AT, accessory tooth				0.8891
	LT, lateral tooth	CV, cardiopyloric valve				0.3842
	LT, lateral tooth	AT, accessory tooth				0.3486
	LcO, lateral cardiac ossicle	PtO, pterocardiac ossicle				0.1008
	PtO, pterocardiac ossicle	CV, cardiopyloric valve				0.0242*
	PtO, pterocardiac ossicle	AT, accessory tooth				0.0316*
	LcO, lateral cardiac ossicle	CV, cardiopyloric valve				0.0057*
	LcO, lateral cardiac ossicle	AT, accessory tooth				0.0050*
	PtO, pterocardiac ossicle	LT, lateral tooth				0.0341*
	ZO, zygocardiac ossicle	MT, medial tooth				0.0115*
	LcO, lateral cardiac ossicle	LT, lateral tooth				0.0022*
	M, membrane	CV, cardiopyloric valve				<.0001*
	M, membrane	AT, accessory tooth				<.0001*
	PtO, pterocardiac ossicle	MT, medial tooth				<.0001*
	LcO, lateral cardiac ossicle	MT, medial tooth				<.0001*
	M, membrane	LT, lateral tooth				<.0001*
	M, membrane	MT, medial tooth				<.0001*
**H**	MT, medial tooth	CV, cardiopyloric valve	89.8181	6	<.0001*	<.0001*
	LT, lateral tooth	CV, cardiopyloric valve				<.0001*
	MT, medial tooth	AT, accessory tooth				0.0002*
	MT, medial tooth	LT, lateral tooth				0.0015*
	LcO, lateral cardiac ossicle	PtO, pterocardiac ossicle				<.0001*
	LcO, lateral cardiac ossicle	CV, cardiopyloric valve				<.0001*
	LT, lateral tooth	AT, accessory tooth				0.0697
	LcO, lateral cardiac ossicle	AT, accessory tooth				0.4397
	PtO, pterocardiac ossicle	CV, cardiopyloric valve				0.4437
	ZO, zygocardiac ossicle	CV, cardiopyloric valve				0.2855
	LcO, lateral cardiac ossicle	LT, lateral tooth				0.3119
	ZO, zygocardiac ossicle	PtO, pterocardiac ossicle				0.0274*
	PtO, pterocardiac ossicle	AT, accessory tooth				0.0104*
	CV, cardiopyloric valve	AT, accessory tooth				0.0057*
	ZO, zygocardiac ossicle	AT, accessory tooth				0.0004*
	ZO, zygocardiac ossicle	LcO, lateral cardiac ossicle				<.0001*
	LcO, lateral cardiac ossicle	MT, medial tooth				<.0001*
	PtO, pterocardiac ossicle	LT, lateral tooth				<.0001*
	ZO, zygocardiac ossicle	LT, lateral tooth				<.0001*
	ZO, zygocardiac ossicle	MT, medial tooth				<.0001*
	PtO, pterocardiac ossicle	MT, medial tooth				<.0001*
**K**	ZO, zygocardiac ossicle	S, setae	72.5666	8	<.0001*	<.0001*
	LcO, lateral cardiac ossicle	S, setae				<.0001*
	PtO, pterocardiac ossicle	M, membrane				0.0056*
	ZO, zygocardiac ossicle	M, membrane				0.0488*
	LcO, lateral cardiac ossicle	M, membrane				0.0878
	PtO, pterocardiac ossicle	LT, lateral tooth				0.7057
	PtO, pterocardiac ossicle	MT, medial tooth				0.8276
	MT, medial tooth	LT, lateral tooth				0.9667
	ZO, zygocardiac ossicle	LcO, lateral cardiac ossicle				0.9629
	PtO, pterocardiac ossicle	CV, cardiopyloric valve				0.7433
	CV, cardiopyloric valve	AT, accessory tooth				0.7265
	LcO, lateral cardiac ossicle	CV, cardiopyloric valve				0.7269
	MT, medial tooth	CV, cardiopyloric valve				0.7414

	PtO, pterocardiac ossicle	AT, accessory tooth				0.5703
	ZO, zygocardiac ossicle	PtO, pterocardiac ossicle				0.4994
	LcO, lateral cardiac ossicle	PtO, pterocardiac ossicle				0.5083
	ZO, zygocardiac ossicle	MT, medial tooth				0.6671
	ZO, zygocardiac ossicle	CV, cardiopyloric valve				0.4177
	LcO, lateral cardiac ossicle	LT, lateral tooth				0.6388
	ZO, zygocardiac ossicle	LT, lateral tooth				0.6523
	LcO, lateral cardiac ossicle	MT, medial tooth				0.5977
	LcO, lateral cardiac ossicle	AT, accessory tooth				0.3451
	LT, lateral tooth	CV, cardiopyloric valve				0.5212
	ZO, zygocardiac ossicle	AT, accessory tooth				0.2743
	MT, medial tooth	AT, accessory tooth				0.4714
	LT, lateral tooth	AT, accessory tooth				0.3655
	M, membrane	CV, cardiopyloric valve				0.0063*
	M, membrane	AT, accessory tooth				0.0028*
	M, membrane	MT, medial tooth				0.0020*
	M, membrane	LT, lateral tooth				0.0014*
	S, setae	M, membrane				<.0001*
	S, setae	CV, cardiopyloric valve				<.0001*
	S, setae	AT, accessory tooth				<.0001*
	S, setae	PtO, pterocardiac ossicle				<.0001*
	S, setae	MT, medial tooth				<.0001*
	S, setae	LT, lateral tooth				<.0001*
**Mg**	LcO, lateral cardiac ossicle	S, setae	125.6214	8	<.0001*	<.0001*
	LcO, lateral cardiac ossicle	M, membrane				<.0001*
	ZO, zygocardiac ossicle	S, setae				<.0001*
	LcO, lateral cardiac ossicle	LT, lateral tooth				0.0001*
	PtO, pterocardiac ossicle	M, membrane				<.0001*
	ZO, zygocardiac ossicle	M, membrane				<.0001*
	LcO, lateral cardiac ossicle	MT, medial tooth				0.0124*
	PtO, pterocardiac ossicle	LT, lateral tooth				0.0191*
	CV, cardiopyloric valve	AT, accessory tooth				0.0015*
	PtO, pterocardiac ossicle	MT, medial tooth				0.0928
	LcO, lateral cardiac ossicle	AT, accessory tooth				0.0234*
	PtO, pterocardiac ossicle	AT, accessory tooth				0.1996
	MT, medial tooth	LT, lateral tooth				0.5930
	LcO, lateral cardiac ossicle	PtO, pterocardiac ossicle				0.8800
	ZO, zygocardiac ossicle	LT, lateral tooth				0.9522
	PtO, pterocardiac ossicle	CV, cardiopyloric valve				0.5299
	ZO, zygocardiac ossicle	MT, medial tooth				0.6895
	MT, medial tooth	AT, accessory tooth				0.6218
	LcO, lateral cardiac ossicle	CV, cardiopyloric valve				0.2956
	ZO, zygocardiac ossicle	AT, accessory tooth				0.2955
	ZO, zygocardiac ossicle	PtO, pterocardiac ossicle				0.1039
	ZO, zygocardiac ossicle	LcO, lateral cardiac ossicle				0.0081*
	LT, lateral tooth	AT, accessory tooth				0.1008
	ZO, zygocardiac ossicle	CV, cardiopyloric valve				0.0010*
	MT, medial tooth	CV, cardiopyloric valve				0.0040*
	S, setae	M, membrane				0.0001*
	M, membrane	CV, cardiopyloric valve				<.0001*
	M, membrane	AT, accessory tooth				<.0001*
	S, setae	CV, cardiopyloric valve				<.0001*
	S, setae	PtO, pterocardiac ossicle				<.0001*
	S, setae	AT, accessory tooth				<.0001*
	LT, lateral tooth	CV, cardiopyloric valve				<.0001*
	M, membrane	MT, medial tooth				<.0001*
	M, membrane	LT, lateral tooth				<.0001*
	S, setae	MT, medial tooth				<.0001*
	S, setae	LT, lateral tooth				<.0001*
**Na**	PtO, pterocardiac ossicle	LT, lateral tooth	34.7796	8	<.0001*	0.0008*
	PtO, pterocardiac ossicle	MT, medial tooth				0.0031*
	ZO, zygocardiac ossicle	LT, lateral tooth				0.0068*
	ZO, zygocardiac ossicle	MT, medial tooth				0.0250*
	PtO, pterocardiac ossicle	M, membrane				0.0892
	ZO, zygocardiac ossicle	S, setae				0.0385*
	M, membrane	LT, lateral tooth				0.0921
	ZO, zygocardiac ossicle	LcO, lateral cardiac ossicle				0.1581
	M, membrane	MT, medial tooth				0.2388
	PtO, pterocardiac ossicle	CV, cardiopyloric valve				0.2718
	ZO, zygocardiac ossicle	M, membrane				0.2886
	ZO, zygocardiac ossicle	CV, cardiopyloric valve				0.5691

	LcO, lateral cardiac ossicle	LT, lateral tooth				0.4680
	MT, medial tooth	LT, lateral tooth				0.5681
	LcO, lateral cardiac ossicle	S, setae				0.7100
	LcO, lateral cardiac ossicle	MT, medial tooth				0.8076
	S, setae	LT, lateral tooth				0.7609
	S, setae	MT, medial tooth				0.8544
	M, membrane	CV, cardiopyloric valve				0.6956
	LcO, lateral cardiac ossicle	M, membrane				0.5413
	ZO, zygocardiac ossicle	PtO, pterocardiac ossicle				0.6003
	PtO, pterocardiac ossicle	AT, accessory tooth				0.6181
	LcO, lateral cardiac ossicle	CV, cardiopyloric valve				0.3872
	S, setae	M, membrane				0.2408
	S, setae	CV, cardiopyloric valve				0.1551
	ZO, zygocardiac ossicle	AT, accessory tooth				0.3029
	MT, medial tooth	CV, cardiopyloric valve				0.1460
	LT, lateral tooth	CV, cardiopyloric valve				0.0518
	CV, cardiopyloric valve	AT, accessory tooth				0.1042
	LcO, lateral cardiac ossicle	PtO, pterocardiac ossicle				0.0599
	LcO, lateral cardiac ossicle	AT, accessory tooth				0.0132*
	S, setae	PtO, pterocardiac ossicle				0.0089*
	M, membrane	AT, accessory tooth				0.0148*
	S, setae	AT, accessory tooth				0.0008*
	MT, medial tooth	AT, accessory tooth				0.0001*
	LT, lateral tooth	AT, accessory tooth				<.0001*
**P**	MT, medial tooth	LT, lateral tooth	163.2894	8	<.0001*	<.0001*
	LcO, lateral cardiac ossicle	LT, lateral tooth				<.0001*
	PtO, pterocardiac ossicle	LT, lateral tooth				<.0001*
	ZO, zygocardiac ossicle	LT, lateral tooth				<.0001*
	LcO, lateral cardiac ossicle	S, setae				<.0001*
	PtO, pterocardiac ossicle	M, membrane				<.0001*
	LcO, lateral cardiac ossicle	M, membrane				<.0001*
	ZO, zygocardiac ossicle	S, setae				<.0001*
	ZO, zygocardiac ossicle	M, membrane				<.0001*
	CV, cardiopyloric valve	AT, accessory tooth				<.0001*
	MT, medial tooth	AT, accessory tooth				0.0206*
	LcO, lateral cardiac ossicle	AT, accessory tooth				0.0021*
	PtO, pterocardiac ossicle	AT, accessory tooth				0.0079*
	ZO, zygocardiac ossicle	AT, accessory tooth				0.0816
	LcO, lateral cardiac ossicle	MT, medial tooth				0.3534
	PtO, pterocardiac ossicle	MT, medial tooth				0.4568
	LcO, lateral cardiac ossicle	PtO, pterocardiac ossicle				0.7486
	S, setae	M, membrane				0.4886
	ZO, zygocardiac ossicle	PtO, pterocardiac ossicle				0.0993
	ZO, zygocardiac ossicle	MT, medial tooth				0.2633
	ZO, zygocardiac ossicle	LcO, lateral cardiac ossicle				0.0190*
	ZO, zygocardiac ossicle	CV, cardiopyloric valve				<.0001*
	LcO, lateral cardiac ossicle	CV, cardiopyloric valve				0.0001*
	PtO, pterocardiac ossicle	CV, cardiopyloric valve				<.0001*
	M, membrane	LT, lateral tooth				0.0057*
	M, membrane	CV, cardiopyloric valve				<.0001*
	M, membrane	AT, accessory tooth				<.0001*
	S, setae	LT, lateral tooth				0.0001*
	S, setae	CV, cardiopyloric valve				<.0001*
	S, setae	AT, accessory tooth				<.0001*
	S, setae	PtO, pterocardiac ossicle				<.0001*
	LT, lateral tooth	AT, accessory tooth				<.0001*
	MT, medial tooth	CV, cardiopyloric valve				<.0001*
	LT, lateral tooth	CV, cardiopyloric valve				<.0001*
	M, membrane	MT, medial tooth				<.0001*
	S, setae	MT, medial tooth				<.0001*
**S**	LcO, lateral cardiac ossicle	LT, lateral tooth	107.9259	8	<.0001*	<.0001*
	LcO, lateral cardiac ossicle	S, setae				<.0001*
	PtO, pterocardiac ossicle	LT, lateral tooth				<.0001*
	ZO, zygocardiac ossicle	S, setae				<.0001*
	LcO, lateral cardiac ossicle	MT, medial tooth				0.0002*
	PtO, pterocardiac ossicle	MT, medial tooth				0.0034*
	LcO, lateral cardiac ossicle	M, membrane				0.0003*
	CV, cardiopyloric valve	AT, accessory tooth				0.0002*
	PtO, pterocardiac ossicle	M, membrane				0.0018*
	LcO, lateral cardiac ossicle	AT, accessory tooth				0.0049*
	MT, medial tooth	LT, lateral tooth				0.1628

	PtO, pterocardiac ossicle	AT, accessory tooth				0.0343*
	ZO, zygocardiac ossicle	LT, lateral tooth				0.4120
	ZO, zygocardiac ossicle	M, membrane				0.4006
	LcO, lateral cardiac ossicle	PtO, pterocardiac ossicle				0.5839
	ZO, zygocardiac ossicle	MT, medial tooth				0.9203
	M, membrane	LT, lateral tooth				0.8875
	ZO, zygocardiac ossicle	AT, accessory tooth				0.2446
	MT, medial tooth	AT, accessory tooth				0.3399
	M, membrane	MT, medial tooth				0.2747
	LcO, lateral cardiac ossicle	CV, cardiopyloric valve				0.0664
	PtO, pterocardiac ossicle	CV, cardiopyloric valve				0.0365*
	M, membrane	AT, accessory tooth				0.0791
	ZO, zygocardiac ossicle	PtO, pterocardiac ossicle				0.0081*
	ZO, zygocardiac ossicle	LcO, lateral cardiac ossicle				0.0010*
	ZO, zygocardiac ossicle	CV, cardiopyloric valve				0.0002*
	LT, lateral tooth	AT, accessory tooth				0.0275*
	M, membrane	CV, cardiopyloric valve				<.0001*
	S, setae	CV, cardiopyloric valve				<.0001*
	S, setae	M, membrane				<.0001*
	S, setae	PtO, pterocardiac ossicle				<.0001*
	S, setae	AT, accessory tooth				<.0001*
	MT, medial tooth	CV, cardiopyloric valve				<.0001*
	LT, lateral tooth	CV, cardiopyloric valve				<.0001*
	S, setae	MT, medial tooth				<.0001*
	S, setae	LT, lateral tooth				<.0001*
**Si**	MT, medial tooth	CV, cardiopyloric valve	68.7669	8	<.0001*	0.0044*
	LT, lateral tooth	CV, cardiopyloric valve				0.0048*
	ZO, zygocardiac ossicle	S, setae				<.0001*
	ZO, zygocardiac ossicle	M, membrane				0.0002*
	PtO, pterocardiac ossicle	M, membrane				0.0005*
	ZO, zygocardiac ossicle	LcO, lateral cardiac ossicle				0.0079*
	PtO, pterocardiac ossicle	CV, cardiopyloric valve				0.0876
	ZO, zygocardiac ossicle	CV, cardiopyloric valve				0.0790
	LcO, lateral cardiac ossicle	S, setae				0.2021
	LcO, lateral cardiac ossicle	M, membrane				0.2405
	MT, medial tooth	LT, lateral tooth				0.9586
	ZO, zygocardiac ossicle	PtO, pterocardiac ossicle				0.9396
	S, setae	M, membrane				1.0000
	LT, lateral tooth	AT, accessory tooth				0.9931
	MT, medial tooth	AT, accessory tooth				0.9314
	LcO, lateral cardiac ossicle	CV, cardiopyloric valve				0.3872
	M, membrane	CV, cardiopyloric valve				0.0470*
	S, setae	CV, cardiopyloric valve				0.0317*
	LcO, lateral cardiac ossicle	PtO, pterocardiac ossicle				0.0115*
	ZO, zygocardiac ossicle	AT, accessory tooth				0.0276*
	PtO, pterocardiac ossicle	AT, accessory tooth				0.0099*
	ZO, zygocardiac ossicle	LT, lateral tooth				0.0841
	CV, cardiopyloric valve	AT, accessory tooth				0.0023*
	ZO, zygocardiac ossicle	MT, medial tooth				0.0770
	S, setae	PtO, pterocardiac ossicle				0.0002*
	PtO, pterocardiac ossicle	LT, lateral tooth				0.0402*
	PtO, pterocardiac ossicle	MT, medial tooth				0.0346*
	LcO, lateral cardiac ossicle	AT, accessory tooth				0.0002*
	M, membrane	AT, accessory tooth				<.0001*
	S, setae	AT, accessory tooth				<.0001*
	LcO, lateral cardiac ossicle	LT, lateral tooth				0.0003*
	LcO, lateral cardiac ossicle	MT, medial tooth				0.0003*
	M, membrane	LT, lateral tooth				<.0001*
	M, membrane	MT, medial tooth				<.0001*
	S, setae	LT, lateral tooth				<.0001*
	S, setae	MT, medial tooth				<.0001*
**E**	MT, medial tooth	CV, cardiopyloric valve	89.3089	6	<.0001*	<.0001*
	LT, lateral tooth	CV, cardiopyloric valve				<.0001*
	MT, medial tooth	AT, accessory tooth				0.0002*
	MT, medial tooth	LT, lateral tooth				0.0020*
	LcO, lateral cardiac ossicle	PtO, pterocardiac ossicle				<.0001*
	LcO, lateral cardiac ossicle	CV, cardiopyloric valve				0.0001*
	LT, lateral tooth	AT, accessory tooth				0.0359*
	PtO, pterocardiac ossicle	CV, cardiopyloric valve				0.3904
	LcO, lateral cardiac ossicle	AT, accessory tooth				0.4624
	ZO, zygocardiac ossicle	CV, cardiopyloric valve				0.2366

	LcO, lateral cardiac ossicle	LT, lateral tooth				0.3119
	ZO, zygocardiac ossicle	PtO, pterocardiac ossicle				0.0601
	PtO, pterocardiac ossicle	AT, accessory tooth				0.0176*
	CV, cardiopyloric valve	AT, accessory tooth				0.0066*
	ZO, zygocardiac ossicle	AT, accessory tooth				0.0005*
	ZO, zygocardiac ossicle	LcO, lateral cardiac ossicle				<.0001*
	LcO, lateral cardiac ossicle	MT, medial tooth				0.0001*
	PtO, pterocardiac ossicle	LT, lateral tooth				<.0001*
	ZO, zygocardiac ossicle	LT, lateral tooth				<.0001*
	ZO, zygocardiac ossicle	MT, medial tooth				<.0001*
	PtO, pterocardiac ossicle	MT, medial tooth				<.0001*
**Zn**	MT, medial tooth	LT, lateral tooth	135.6499	8	<.0001*	<.0001*
	LcO, lateral cardiac ossicle	S, setae				<.0001*
	PtO, pterocardiac ossicle	M, membrane				<.0001*
	LcO, lateral cardiac ossicle	M, membrane				<.0001*
	ZO, zygocardiac ossicle	S, setae				<.0001*
	ZO, zygocardiac ossicle	M, membrane				<.0001*
	LcO, lateral cardiac ossicle	LT, lateral tooth				0.0010*
	MT, medial tooth	AT, accessory tooth				0.0022*
	PtO, pterocardiac ossicle	LT, lateral tooth				0.0409*
	LcO, lateral cardiac ossicle	AT, accessory tooth				0.0102*
	CV, cardiopyloric valve	AT, accessory tooth				0.0136*
	ZO, zygocardiac ossicle	LT, lateral tooth				0.1800
	PtO, pterocardiac ossicle	AT, accessory tooth				0.0927
	LcO, lateral cardiac ossicle	PtO, pterocardiac ossicle				0.1799
	ZO, zygocardiac ossicle	AT, accessory tooth				0.2738
	MT, medial tooth	CV, cardiopyloric valve				0.5489
	S, setae	M, membrane				0.9260
	LcO, lateral cardiac ossicle	CV, cardiopyloric valve				0.7274
	ZO, zygocardiac ossicle	PtO, pterocardiac ossicle				0.6748
	LT, lateral tooth	AT, accessory tooth				0.6631
	PtO, pterocardiac ossicle	CV, cardiopyloric valve				0.2742
	ZO, zygocardiac ossicle	CV, cardiopyloric valve				0.2236
	ZO, zygocardiac ossicle	LcO, lateral cardiac ossicle				0.2266
	LcO, lateral cardiac ossicle	MT, medial tooth				0.0598
	ZO, zygocardiac ossicle	MT, medial tooth				0.0266*
	PtO, pterocardiac ossicle	MT, medial tooth				0.0102*
	LT, lateral tooth	CV, cardiopyloric valve				0.0041*
	M, membrane	CV, cardiopyloric valve				<.0001*
	S, setae	CV, cardiopyloric valve				<.0001*
	M, membrane	AT, accessory tooth				<.0001*
	S, setae	AT, accessory tooth				<.0001*
	S, setae	PtO, pterocardiac ossicle				<.0001*
	M, membrane	MT, medial tooth				<.0001*
	M, membrane	LT, lateral tooth				<.0001*
	S, setae	MT, medial tooth				<.0001*
	S, setae	LT, lateral tooth				<.0001*

Supplementary Table 3. Correlation coefficients between the parameters (elemental content and mechanical properties), estimated by row-wise method. All structures and cuticle layers are pooled together.

	Ae	Ca	Cl	F	Fe	H	K	Mg	Na	P	S	Si	E	Zn
**Ae**	1.0000	0.8674	0.6912	0.6917	0.6037	0.7883	0.4715	0.4510	0.0724	0.7433	0.1998	0.7941	0.7624	0.5565
**Ca**	-	1.0000	0.7474	0.6510	0.6010	0.4689	0.4661	0.4982	0.0836	0.7745	0.2092	0.4359	0.4489	0.5023
**Cl**	-	-	1.0000	0.5695	0.4453	0.3192	0.4179	0.4018	0.0254	0.5631	0.1237	0.3267	0.3132	0.4330
**F**	-	-	-	1.0000	0.4703	0.4446	0.3600	0.5348	-0.0479	0.5103	0.3106	0.3529	0.4188	0.5112
**Fe**	-	-	-	-	1.0000	0.3094	0.2123	0.2242	0.0353	0.4579	0.0178	0.3335	0.2759	0.6441
**H**	-	-	-	-	-	1.0000	0.1411	-0.0902	-0.0636	0.1022	-0.3074	0.7953	0.9875	0.1995
**K**	-	-	-	-	-	-	1.0000	0.3273	0.1071	0.5114	0.2525	0.2322	0.1413	0.3149
**Mg**	-	-	-	-	-	-	-	1.0000	0.0824	0.4740	0.4134	0.1076	-0.0979	0.4001
**Na**	-	-	-	-	-	-	-	-	1.0000	0.1269	0.0495	0.0340	-0.0634	-0.0113
**P**	-	-	-		-	-	-	-	-	1.0000	0.2928	0.3310	0.1238	0.4125
**S**	-	-	-	-	-	--	-	-	-	-	1.0000	-0.0166	-0.3178	0.3599
**Si**	-	-	-	-	-	-	-	-	-	-	-	1.0000	0.7699	0.3205
**E**	-	-	-	-	-	-	-	-	-	-	-	-	1.0000	0.1737
**Zn**	-	-	-	-	-	-	-	-	-	-	-	-	-	1.0000

Supplementary Table 4. Correlation coefficients between the parameters (elemental content and mechanical properties), estimated by row-wise method. The data are for the epicuticle only. All structures are pooled together.

	Ae	Ca	Cl	F	Fe	H	K	Mg	Na	P	S	Si	E	Zn
**Ae**	1.0000	0.8466	0.5818	0.5761	0.6055	0.7446	0.4237	0.2587	0.0027	0.6529	0.1128	0.8908	0.7032	0.5032
**Ca**	-	1.0000	0.5794	0.5536	0.5798	0.4993	0.4514	0.3582	0.0596	0.7563	-0.1001	0.5499	0.4687	0.3919
**Cl**	-	-	1.0000	0.3854	0.3818	0.3224	0.4561	0.1456	-0.0198	0.4713	-0.1989	0.3864	0.3110	0.2504
**F**	-	-	-	1.0000	0.4525	0.3918	0.3000	0.4894	-0.1443	0.3254	0.0395	0.3549	0.3374	0.3567
**Fe**	-	-	-	-	1.0000	0.2928	0.3084	0.1502	0.0349	0.4447	-0.0110	0.4286	0.2419	0.6787
**H**	-	-	-	-	-	1.0000	0.1007	-0.1371	-0.1780	-0.0655	-0.3717	0.7757	0.9825	0.1878
**K**	-	-	-	-	-	-	1.0000	0.2096	0.1050	0.5170	0.0788	0.2210	0.0912	0.3645
**Mg**	-	-	-	-	-	-	-	1.0000	0.0760	0.2989	0.0437	0.0385	-0.1534	0.1424
**Na**	-	-	-	-	-	-	-	-	1.0000	0.1246	0.0237	-0.0401	-0.1835	-0.0509
**P**	-	-	-		-	-	-	-	-	1.0000	0.0208	0.3444	-0.0373	0.2937
**S**	-	-	-	-	-	--	-	-	-	-	1.0000	-0.1660	-0.3950	0.2185
**Si**	-	-	-	-	-	-	-	-	-	-	-	1.0000	0.7383	0.4081
**E**	-	-	-	-	-	-	-	-	-	-	-	-	1.0000	0.1408
**Zn**	-	-	-	-	-	-	-	-	-	-	-	-	-	1.0000

Supplementary Table 5. Correlation coefficients between the parameters (elemental content and mechanical properties), estimated by row-wise method. The data are for the exocuticle only. All structures are pooled together.

	Ae	Ca	Cl	F	Fe	H	K	Mg	Na	P	S	Si	E	Zn
**Ae**	1.0000	0.9282	0.7168	0.6453	0.7181	0.5052	0.0745	0.3410	0.0828	0.7509	0.0592	0.1006	0.5133	0.4878
**Ca**	-	1.0000	0.7085	0.4547	0.6212	0.3347	0.0522	0.1966	-0.0521	0.5843	-0.0714	0.1678	0.3239	0.3479
**Cl**	-	-	1.0000	0.4466	0.5213	0.3733	-0.0575	0.1929	-0.1007	0.2983	-0.1616	0.0494	0.3819	0.3372
**F**	-	-	-	1.0000	0.4801	0.4442	-0.0144	0.2397	-0.2081	0.3244	0.1717	-0.1792	0.4596	0.4716
**Fe**	-	-	-	-	1.0000	0.3750	0.0753	0.3268	-0.0494	0.4619	0.0537	0.0319	0.3656	0.5987
**H**	-	-	-	-	-	1.0000	-0.0827	-0.0779	-0.1120	0.0825	-0.3094	0.0079	0.9844	0.4301
**K**	-	-	-	-	-	-	1.0000	-0.0225	0.0211	0.1608	-0.0110	0.0989	-0.0679	-0.0989
**Mg**	-	-	-	-	-	-	-	1.0000	0.0149	0.2611	0.4180	0.1943	-0.0740	0.3910
**Na**	-	-	-	-	-	-	-	-	1.0000	-0.0117	0.0759	0.3661	-0.0997	-0.0275
**P**	-	-	-		-	-	-	-	-	1.0000	0.1061	0.1000	0.0948	0.2408
**S**	-	-	-	-	-	--	-	-	-	-	1.0000	-0.0580	-0.3065	0.2086
**Si**	-	-	-	-	-	-	-	-	-	-	-	1.0000	0.0324	-0.0126
**E**	-	-	-	-	-	-	-	-	-	-	-	-	1.0000	0.4579
**Zn**	-	-	-	-	-	-	-	-	-	-	-	-	-	1.0000

Supplementary Table 6. Correlation coefficients between the parameters (elemental content), estimated by row-wise method. The data are for the setae only.

	Si	F	Na	Mg	P	S	Cl	K	Ca	Zn	Ae
**Si**	1.0000	0.0000	0.0000	0.0000	0.0000	0.0000	0.0000	0.0000	0.0000	0.0000	0.0000
**F**	-	1.0000	-0.0656	0.0764	0.5527	0.4543	-0.2326	-0.0499	0.4440	0.0556	0.7154
**Na**	-	-	1.0000	-0.0902	0.1309	-0.1337	0.2699	-0.0956	-0.1003	-0.0574	-0.1171
**Mg**	-	-	-	1.0000	-0.2867	0.3226	-0.1428	0.2016	0.3348	0.0265	0.3667
**P**	-	-	-	-	1.0000	0.2764	-0.1907	-0.0900	0.2856	-0.0659	0.4568
**S**	-	-	-	-	-	1.0000	-0.0690	-0.1512	0.2933	-0.2071	0.4151
**Cl**	-	-	-	-	-	-	1.0000	-0.1514	-0.2081	-0.0909	-0.1922
**K**	-	-	-	-	-	-	-	1.0000	-0.1459	0.4781	0.0358
**Ca**	-	-	-	-	-	-	-	-	1.0000	-0.2439	0.8546
**Zn**	-	-	-	-	-	--	-	-	-	1.0000	-0.0631
**Ae**	-	-	-	-	-	-	-	-	-	-	1.0000
